# Bariatric Surgery and Inflammatory Bowel Disease: National Trends and Outcomes Associated with Procedural Sleeve Gastrectomy vs Historical Bariatric Surgery Among US Hospitalized Patients 2009–2020

**DOI:** 10.1007/s11695-023-06833-7

**Published:** 2023-10-07

**Authors:** Joseph-Kevin Igwe, Phani Keerthi Surapaneni, Erin Cruz, Cedric Cole, Kingsley Njoku, Jisoo Kim, Ugo Alaribe, Kelechi Weze, Bilal Mohammed

**Affiliations:** 1 School of Medicine, Department of Medicine, Stanford University, 291 Campus Drive, Stanford, CA 94305 USA; 2 Department of Medicine, Morehouse School of Medicine, 720 Westview Dr. SW, Atlanta, GA 30313 USA; 3 American Heart Association Strategically Focused Research Network on the Science of Diversity in Clinical Trials Research Fellowship, 5001 S Miami Blvd #300, Durham, NC 27703 USA; 4 Department of Internal Medicine, WVU of Medicine, Morgantown, USA; 5 Department of Medicine, Emory University School of Medicine, Atlanta, USA; 6 Department of Surgery, Texas Tech University Health Sciences Center at El Paso, El Paso, USA; 7 School of Medicine, Caribbean Medical University, Willemstad, USA; 8 Department of Medicine, Ascension Saint Vincent, Indianapolis, USA

**Keywords:** Bariatric surgery, Irritable bowel disease, Micronutrient deficiency

## Abstract

**Abstract:**

**Purpose:**

The association between bariatric surgery and IBD-related inpatient outcomes is not well characterized. We report, analyze, and compare inpatient trends and outcomes among encounters with a history of bariatric surgery (Hx-MBS) compared to those receiving bariatric surgery during index admission (PR-MBS) admitted from 2009 to 2020.

**Methods:**

Retrospective cohort design: the 2009–2020 National Inpatient Sample (NIS) databases were used to identify hospital encounters with patients aged ≥ 18 years with a history of MBS (Hx-MBS) or with procedure coding indicating MBS procedure (PR-MBS) according to International Classification of Diseases, Ninth (ICD-9-CM/ ICD-9-PCS) or Tenth Revision (ICD-10-CM/ICD-10-PCS) Clinical Modification/Procedure Coding System during index admission (ICD-9-CM: V4586; ICD-10-CM: Z9884; ICD-9-PR: 4382, 4389; ICD-10-PR: 0DB64Z3, 0DB63ZZ). Pearson *χ*2 analysis, analysis of variance, multivariable regression analyses, and propensity matching on independent variables were conducted to analyze significant associations between variables and for primary outcome inflammatory bowel disease-related admission, and secondary outcomes: diagnosis of nonalcoholic steatohepatitis, nonalcoholic fatty liver disease, or chronic mesenteric ischemia during admission.

**Results:**

We identified 3,365,784 (76.20%) Hx-MBS hospitalizations and 1,050,900 hospitalizations with PR-MBS (23.80%). Propensity score matching analysis demonstrated significantly higher odds of inflammatory bowel disease, and chronic mesenteric ischemia for Hx-MBS compared to PR-MBS, and significantly lower odds of nonalcoholic steatohepatitis and nonalcoholic fatty liver disease for Hx-MBS compared to PR-MBS.

**Conclusion:**

In our study, Hx-MBS was associated with significantly increased odds of inflammatory bowel disease and other GI pathologies compared to matched controls. The mechanism by which this occurs is unclear. Additional studies are needed to examine these findings.

**Graphical abstract:**

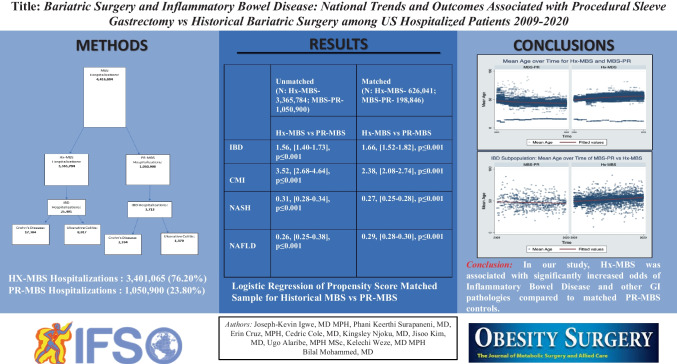

**Supplementary Information:**

The online version contains supplementary material available at 10.1007/s11695-023-06833-7.

## Introduction

### Background

Metabolic and bariatric surgery (MBS) is the mainstay surgical treatment for patients with obesity (body mass index (BMI) > 35 kg/M2) and at least one obesity-related comorbidity [1]. MBS results in decreased available, absorptive surface area within the alimentary tract and decreases total production of available intrinsic factor (IF) for vitamin B_12_ (cobalamin) absorption in the ileum as seen in laparoscopic sleeve gastrectomy (SG) or alters micronutrient and gut bacteria luminal interactions within the gastric mucosa, proximal duodenum, and along the alimentary tract for both Roux-en-Y gastric bypass (RYGB) and SG [1–3]. RYGB is a malabsorption procedure in which a conduit circuit around the duodenum is created resulting in decreased nutrient intake generally, while SG achieves weight loss by removing a portion of the gastric mucosa and limiting or restricting oral intake postoperatively. However, each is associated with similar micronutrient deficiency (MD) profiles, albeit through different mechanisms, and reports from prior studies note that RGYB is associated with similar if not worse MD profile compared to sleeve gastrectomy [1, 4–15]. While MBS has proven efficacious for remission of obesity, diabetes mellitus, hypertension, dyslipidemia, and OSA (Obstructive Sleep Apnea), little research is available on associated complications related to essential mineral and vitamin deficiencies seen as sequelae of the procedure [1].

### The Gut-Associated Lymphoid Tissue (GALT) and the Gut Microbiome

The gut-associated lymphoid tissue (GALT) consists of both isolated and aggregated lymphoid follicles and is one of the largest lymphoid organs, containing up to 70% of the body’s immunocytes. Peyer’s patches (PP) located along the antimesenteric side of the gut with a marked predominance at the distal 25cm of the terminal ileum and demonstrate a Th1 predominant immune profile with greater secretion of IFNγ, TNFα, and interleukin (IL)-2, but low levels of IL-4, IL-6, and IL-10. The follicle-associated epithelium (FAE) is characterized by many B- and T-cells, macrophages, and follicular dendritic cells (DC), with greater porosity than regular epithelium and specialized enterocytes M-cells which are responsible for transcytosis of intact luminal material such as proteins, antigens, bacteria, and viruses. These cells are constantly renewed by precursor cells in adjacent crypt cells and differentiate from enterocytes under the influence of membrane-bound lymphotoxin (LTα1β2) present on local lymphoid cells, mainly B-cells [16]. What is more, the proportional enterocyte-to-M-cell composition of FAE may be modulated by bacteria present in the gut lumen, demonstrating this phenotypic plasticity by increasing the number of M-cells in response to greater pathogen exposure within the gut [16, 17].

The gut microbiome (GM) contributes to energy and fat metabolism through the production of short-chain fatty acids (SCFA): acetate, propionate, and butyrate (accounting for ≥ 95% of SCFA with an approximate molar ratio of 60:20:20 in the colon and stool; with Bacteroidetes (gram negative) producing acetate and propionate; and Firmicute (gram positive) producing butyrate primarily). These SCFA stimulate hepatic synthesis of lipids; participate in hepatic gluconeogenesis and reduction of de-novo fatty acids and cholesterol synthesis; and regulate energy metabolism, modulate insulin sensitivity, and exert anti-inflammatory effects, respectively [18]. The majority of SCFA is absorbed by the host (~ 95%) in exchange for bicarbonate, and, as the concentration of SCFA decline from proximal to distal colon, the pH increases from cecum to rectum, indirectly preventing overgrowth of pH-sensitive pathogenic bacteria such as Enterobacteriaceae and Clostridia and also modulating GM composition [18]. As such, the luminal pH is a balance of SCFA production from GM fermentation and host bicarbonate production. Limited fermentable dietary fibers in the distal large intestine increase the pH and result in decreased butyrate-producing bacteria and acetate- and propionate-producing Bacteroides-related bacteria become dominant [18]. It has been reported that the GM of obese patients has less variability compared to others; a decreased functional capacity for fermentation and production of SCFA; and a decreased Bacteroidetes/Firmicute ratio compared to normal-weight controls [10, 18]. Changes in the GM following MBS are believed to play a role in weight loss [10–12, 14, 18–20].

Moreover, the alteration of gut microbiota has also been associated with IBD (inflammatory bowel disease) presentation, and previous studies have shown intestinal microbiota are key players in the pathogenesis of IBD [1–3, 21–38]. In the PPs of ileal CD compared to controls, Keita et al. demonstrated increased nonpathogenic *E. coli* translocation associated with increased percentage of *E. coli* colocalizing with mature DCs (CD83+CCR7− DC) which are capable of phagocytizing live bacteria [39]. Classically, CD is thought to be driven by abnormal Th1-mediated inflammatory responses induced by dendritic cells that present commensal bacteria [16, 17, 40, 41]. However, recent findings implicate aberrant innate immune responses mediated through innate lymphoid cells, a novel family of effector lymphocytes in IBD that produce IBD-relevant cytokines in response to the gut microbiome, as well as involvement of the type 17 CD4+ T-helper T-cell (Th17)-IL-23 axis as contributing to the pathogenesis of CD [40, 41]. Recent findings also highlight the role of B-cell pathology in ulcerative colitis, noting major perturbations within the mucosal B cell compartment, including an expansion of naive B cells and IgG+ plasma cells with curtailed diversity and maturation [40, 42]. As such, B-cell-related immune dysfunctional pathways reported in IBD may be mediated through PPs’ response to environmental insults and interactions.

### Micronutrients: Vitamin D, Vitamin B_12_, and Others

Pre-operative testing prior to MBS includes vitamin B_12_ and folic acid serum levels, complete blood count, chemistry panel and lipid profile, liver and thyroid function testing, and coagulation tests.

Cobalamin is a key micronutrient integral to immune cell regulation and DNA synthesis, and fatty acid and amino acid metabolism, with no international consensus value but generally agreed upon ranges between 120 and 220 pmol/L (higher threshold in pregnancy) and 650 and 850 pmol/L. [3, 43, 44] Holotranscobalamin, methyl malonic acid, and homocysteine (tHcy) may be useful markers of cobalamin deficiency for those patients with borderline levels (150–220 pmol/L) [44]. The daily recommended intake of cobalamin is 2.4 μg; however, at baseline, humans absorb approximately 2 μg per meal of cobalamin due to limited IF secretion [1, 2, 45]. Cobalamin absorption is dependent upon IF released from parietal cells within the gastric body for absorption; however, microorganisms within the GM are also able to produce cobalamin to a limited extent. Cobalamin is bound by R-factors released from salivary glands and gastric mucosal cells. Pepsinogen released from chief cells within the gastric body is converted to pepsin by proteolytic enzymes released from the pancreas which in turn facilitates the conversion of absorbable, divalent cations in the proximal duodenum, and release of cobalamin from R-factor binders. The IF then complexes with cobalamin for transport to the terminal ileum for systemic absorption. Additionally, the malabsorption of vitamin B9 and B12 following bariatric surgery affects the re-methylation pathway of homocysteine, leading to hyper-homocysteinemia [46–48]. Homocysteine increases TNF expression and increases oxidative stress, inducing a pro-inflammatory vascular state [46, 47], and prior research has demonstrated relationships between GM changes and gastrointestinal oxygen supply [48, 49].

The liver may store a large amount of vitamin B12 and may delay the development of cobalamin deficiency syndrome for several years. When symptomatic, patients may present with megaloblastic anemia and neurological symptoms (paresthesia, unsteady gait, poor memory, agitation, confusion, depression) [1, 2, 13, 45, 50, 51]. Decreased IF is expected following SG due to resection of the fundus and loss of parietal cells which produce IF [3, 11]; and following RGYB due to procedure-related separation of cofactors and decreased surface area for cofactor interactions. Prior research reports lower concentration of IF among MBS patients compared to controls [3, 12, 52].

Vitamin D is a fat-soluble vitamin which modulates the innate and adaptive immune responses. Vitamin D deficiency is associated with increased autoimmunity and susceptibility to infection. Prior research has shown that the immune cells (B cells, T cells, and antigen presenting cells) not only express vitamin D receptors, but also are capable of synthesizing the active vitamin D metabolite, suggesting that it has a functional role that extends beyond just bone and calcium homeostasis [53]. Additionally, previous experimental studies have noted the importance of vitamin D in maintenance of intestinal mucosal integrity and intimate the significant role it may play in cancer prevention [12, 27, 28].

Micronutrient deficiencies have been associated with deficiency anemia: cobalamin, iron, copper, zinc; hypothyroidism: iodine, calcium, copper, iron, and zinc, magnesium etc.; and liver disease/NASH: choline/phosphatidylcholine, vitamin D [1–3, 21–26, 43–45, 54, 55]. As the protective antioxidant effects associated with micronutrients are lost, micronutrient deficiencies translate to both the host and the GM itself. Ciobârcă et al. note Gram-positive bacteria require more magnesium than Gram-negative bacteria and report the reduction of commensal anaerobic bacteria within the two major phyla Bacteroidetes and Firmicutes secondary to reductions in dietary antioxidants [14]. In RYGB, nutrients are separated from biliopancreatic extract and biliary fluid, coming together in the lower part of the intestine and exposing the distal jejunum and proximal ileum to excessive nutrients [10], and has been associated with greater changes in the GM following MBS compared to SG [10, 11, 14]. Divalent cation and vitamin (micronutrient) absorption and mediators that affect the functional interaction of cofactors related to absorption such as quantity of pepsinogen for conversion to pepsin or quantity of IF available for binding of cobalamin as seen in SG; or altered micronutrient and GM interactions with bile acids and other cofactors as in SG and RYGB may play key roles in the incidence and modulation of disease [3, 10, 14, 50–52, 56–68]. However, the role that these micronutrient deficiencies play and their significance in the incidence of IBD and other gastrointestinal pathologies among the bariatric surgery patient population has not been completely elucidated.

### Study Objective

Metabolic and bariatric surgery is associated with pathological conditions related to MD which undergo key cofactor and absorptive interactions within the intestine at which the MBS procedure is directed. Our study focuses on encounters associated with procedural sleeve gastrectomy; however, additional information regarding the study population, types of bariatric surgery (sleeve gastrectomy (SG), adjustable gastric banding (AGB), duodenal switch (DS), Roux-en-Y gastric bypass (RYGB), etc.), and associated metabolic and micronutrient supplementation recommendations can be found in the Online Supplement.

Among patients with IBD, previous studies have noted associations between small bowel surgery, intestinal microbiota, and incidence of NASH (nonalcoholic steatohepatitis) and NAFLD (nonalcoholic fatty liver disease) [9, 10, 25]. Other studies have demonstrated that environmental risk factors in addition to genetic risk factors play a role in the development of IBD, noting nutritional marker differences and nutrition-related genetic variance between IBD patients and non-IBD patients [20–28];as well as alterations in the GM for obese compared to non-obese patients [36, 69–73]. However, the relationship between bariatric surgery, MD, and IBD- and gastrointestinal (GI) disease-related inpatient outcomes is not known. We hypothesized that the altered gastrointestinal ecosystem secondary to MBS and associated with aberrant micronutrient absorption and interactions would increase risk for IBD and associated pathological conditions such as chronic mesenteric ischemia (CMI), NASH, and NAFLD among those with history of bariatric surgery compared to others.

In our study, we compared two similar groups: patients who have a history of receiving bariatric surgery (Hx-MBS) and those who received bariatric surgery during index admission from 2009 to 2020 (PR-MBS). Our study examines the trend and outcomes associated with both groups to better understand the consequences of MBS.

## Methods

### Data Source

The NIS database is the largest publicly available all-payer inpatient healthcare database designed to produce U.S. (United States) regional and national estimates of inpatient utilization, access, charges, quality, and outcomes. The unit of measure in determining incidence was a hospitalization encounter. Each encounter represents one hospitalization.

### Study Population

The 2009 to 2020 National Inpatient Sample (NIS) databases were used to identify encounters with patients aged ≥ 18 years with history of MBS (Hx-MBS) according to International Classification of Diseases, Ninth (ICD-9 CM) or Tenth Revision (ICD-10 CM) Clinical Modification (ICD-9 CM: V4586; ICD-10-CM: Z9884) or with procedure coding indicating MBS procedure (PR-MBS) during index admission according to International Classification of Diseases Ninth (ICD-9-PCS) or Tenth Revision (ICD-10-PCS) Procedure Coding System (Laparoscopic vertical (sleeve) gastrectomy ICD-9 PR: 4382, ICD-10 PR: 0DB64Z3; Open and other partial gastrectomy ICD-9 PR: 4389; Excision of Stomach, Percutaneous Approach: ICD-10 PR:0DB63ZZ), collating observations (patient encounters) into a single dataset. Additional information on the study population can be found in Supplementary material.

### Statistical Analysis

Pearson *χ* [2] analysis, linear regression, and linear and logistic multivariable regression analyses (MRA) and propensity score matching (PSM) were conducted to analyze associations between variables and primary outcome of interest, incidence of IBD-related admission; and secondary outcomes: incidence of CMI, NASH, and NAFLD based on ICD coding in any position of the medical claim during index admission among the inpatient population with and without Hx-MBS. We used a 0.1 caliper width to account for age, race, sex, admission year, hospital characteristics: hospital region, hospital bed size, location/teaching status of hospital; primary insurance payor: Medicare, Medicaid, Self-Pay, Other, No Charge; length of stay (LOS), total charge (TOTCHG), median household income for patient’s ZIP code, population median-Elixhauser-index sum score 1 or more, comorbidities, and long-term medications. STATA/MP software (StataCorp. 2021. Stata Statistical Software: Release 17. College Station, TX: StataCorp LLC) was used for all analyses. Multivariable regression was performed on the matched sample to determine adjusted odds for outcomes of interest.

## Results

**Fig. 1:**
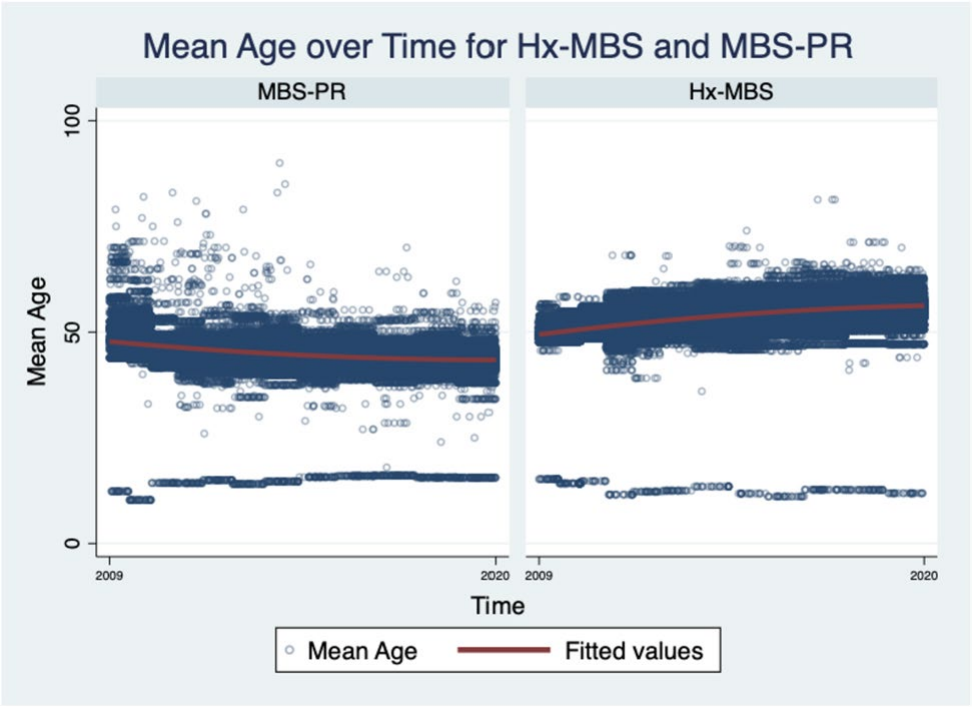
Mean age over time for Hx-MBS and PR-MBS. The mean age of Hx-MBS over the index period was 53.47 ± 0.02. The mean age of PR-MBS over the index period was 44.65 ± 0.05

We identified 3,365,784 (76.20%) patient encounters with Hx-MBS, and 1,050,900 patient encounters with PR-MBS (23.80%) meeting inclusion criteria. The mean age of Hx-MBS was 53.50 ± 0.04 with 78.71% females. The mean age of PR-MBS was 44.68 ± 0.05 with 78.03% females (Figure 1). There were 25,401 (0.75%) admission associated with IBD among the Hx-MBS population, and 3,713 (0.35%) admission associated with IBD among the PR-MBS population. Among those with Hx-MBS and IBD, 17,384 (68.44%) were diagnosed with Crohn’s disease (CD) and 8017 (31.56%) were diagnosed with ulcerative colitis disease (UC) (*p* = 0.002); among those with PR-MBS and IBD, 2334 (62.86%) were diagnosed with Crohn’s disease and 1379 (37.14%) were diagnosed with ulcerative colitis (*p* = 0.002). The mean age of those with IBD among Hx-MBS was 52.08 ± 0.20, and 47.15 ± 0.45 among those with IBD within PR-MBS (*p* ≤ 0.001). The mean age in years of those with NASH or NAFLD was higher for Hx-MBS compared to PR-MBS (51.69 ± 0.13 vs 45.10 ± 0.16, *p* ≤ 0.001; 50.66 ± 0.10 vs 44.43 ± 0.10, *p* ≤ 0.001), and the mean age of those with CMI was lower for Hx-MBS compared to PR-MBS (56.34 ± 0.28 vs 61.49 ± 1.76, *p* ≤ 0.001, respectively).

Cobalamin deficiency, vitamin D deficiency, peptic ulcer disease without bleeding (PUD), deficiency anemia, and peripheral vascular disease (PVD) were significantly associated with a Hx-MBS (Table 1). The mean percentage of patients with chronic PUD was 2.80 percentage points (pp) higher among Hx-MBS than PR-MBS (*p* ≤ 0.001) (Table 1). The mean length of stay (LOS) and total charge (TOTCHG) of the Hx-MBS patients (4.31 ± 0.01 days; $49,160.56 ± 266.62) was significantly different than that of PR-MBS patients (2.08 ± 0.02 days; *p* ≤ 0.001; $52,701.29 ± 471.30) (*p* ≤ 0.001 for each).

**Table 1:**
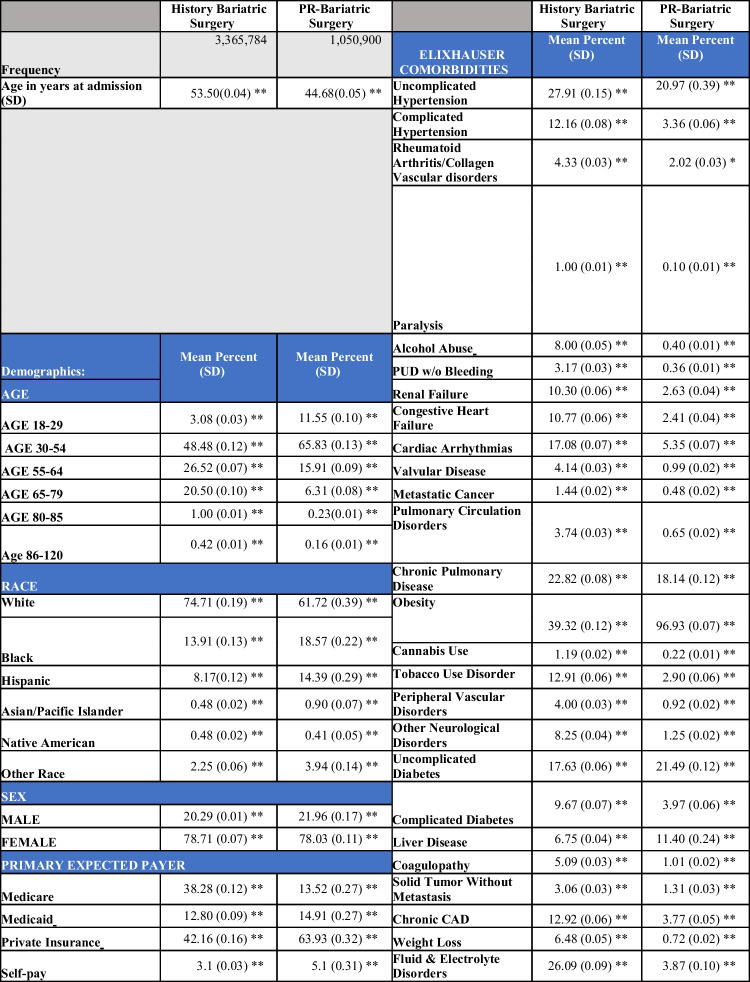

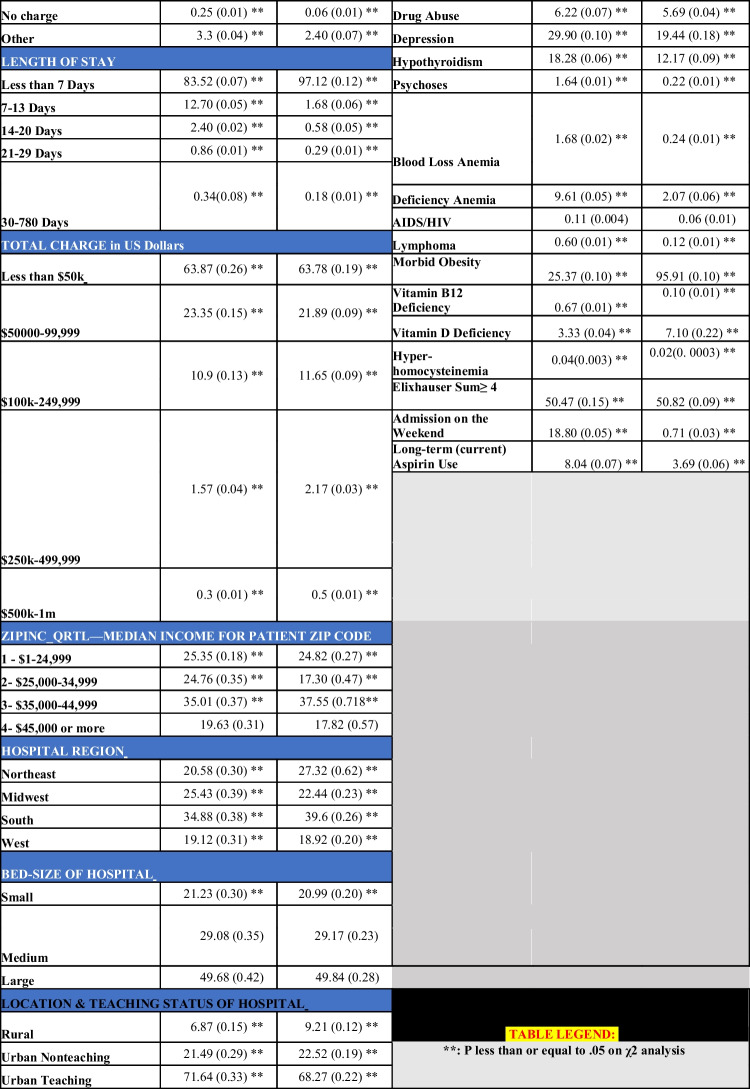
Demographic table

**Fig. 2:**
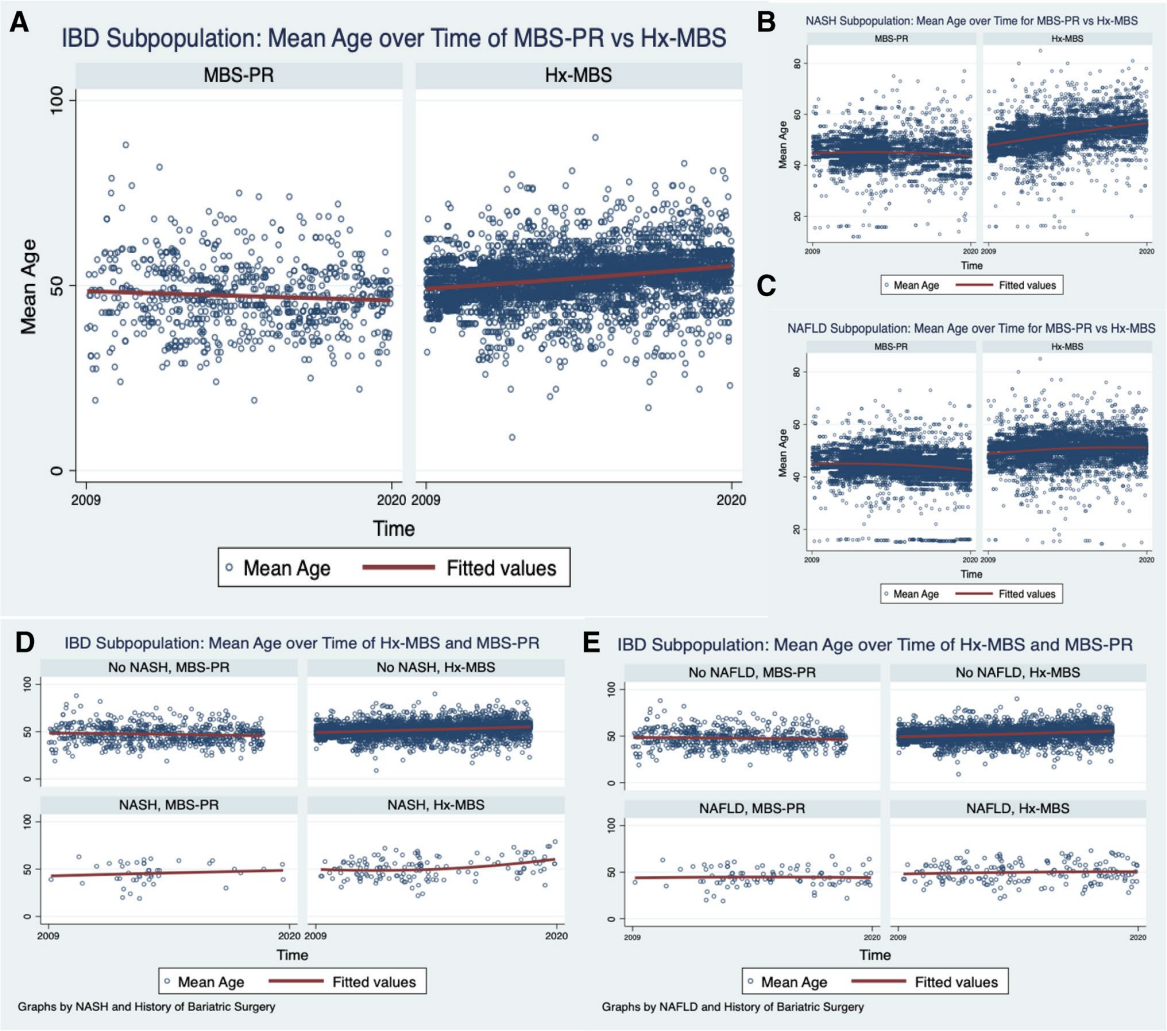
[Read left to right; top to bottom]: (**A**) IBD subpopulation: mean age over time of PR-MBS vs Hx-MBS; (**B**) NASH subpopulation: mean age over time for PR-MBS vs Hx-MBS; (**C**) NAFLD subpopulation: mean age over time for PR-MBS vs Hx-MBS; (**D**) IBD subpopulation: mean age over time by NASH and PR-MBS vs Hx-MBS; (**E**) IBD subpopulation: mean age over time by NAFLD and PR-MBS vs Hx-MBS

**Fig. 3:**
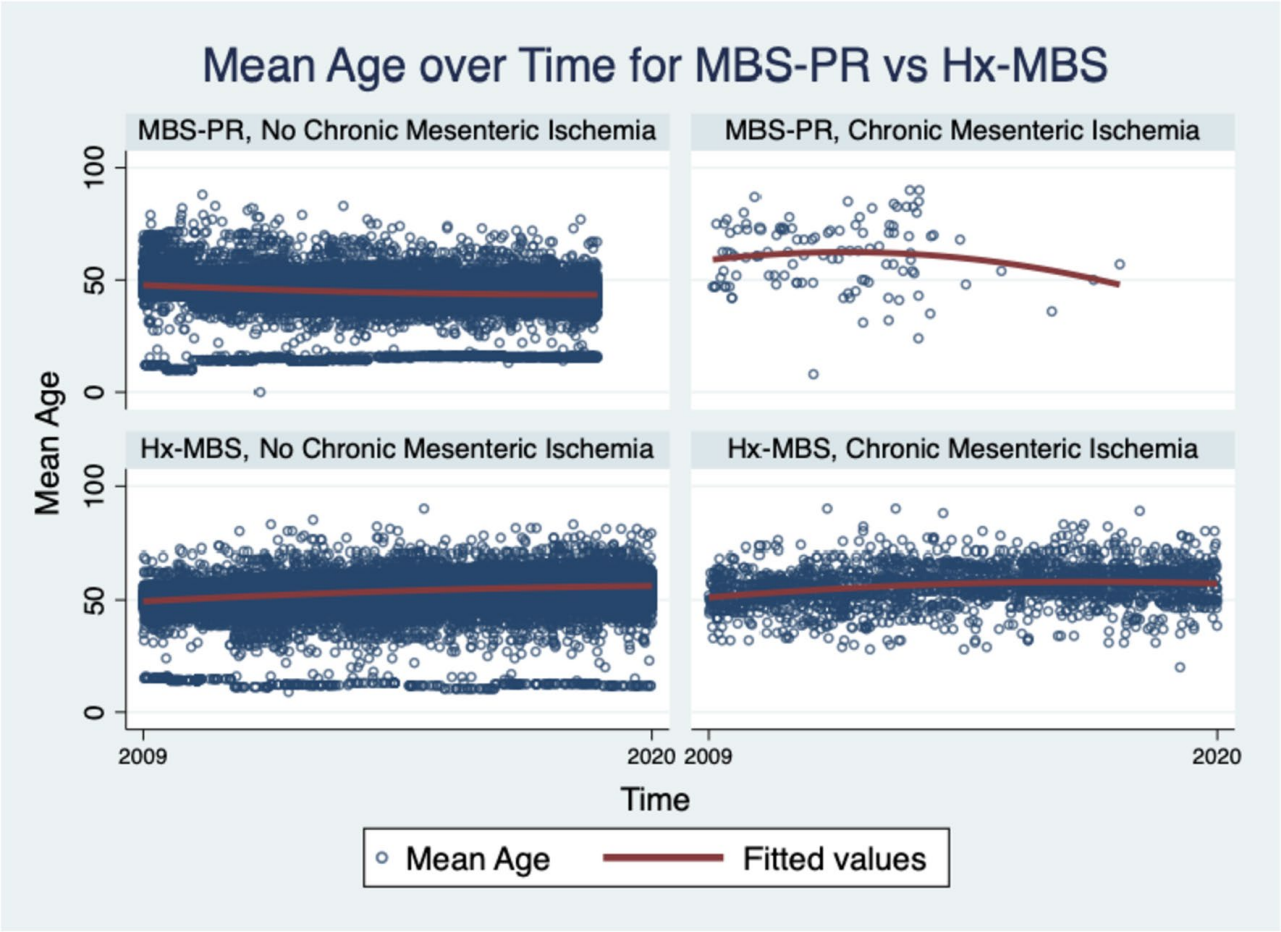
Hx-MBS mean age by chronic mesenteric ischemia over time 2009–2020. Legend: Over the index period, the mean age of those with CMI was 56.34 ± 0.28 for Hx-MBS and 61.49 ± 1.76 for PR-MBS (*p* ≤ 0.001)

The incidence of IBD, CMI, and hypothyroidism among the Hx-MBS population was significantly higher than those among PR-MBS (Table 1) (*p* ≤ 0.001 for each), but the incidence of NASH and NAFLD was significantly lower within the Hx-MBS subpopulation compared to PR-MBS (*p* ≤ 0.001 for both). Additionally, the mean incidence of IBD, hypothyroidism, and CMI was significantly higher for obese and non-obese Hx-MBS patients (0.62%, 0.84%; 18.73%, 17.96%; 0.26%, 0.37%, respectively) compared to obese PR-MBS patients (0.33%; 12.25%; 0.02%) (*p* ≤ 0.05 for each), and there was significantly higher incidence of IBD and CMI for non-obese Hx-MBS patients compared to Hx-MBS obese patients (*p* ≤ 0.001). Among those with NAFLD or NASH, there was a higher incidence of IBD among Hx-MBS patients compared to PR-MBS (1.14% vs 0.44%, *p* ≤ 0.001; 1.33% vs 0.46%, *p* ≤ 0.001, respectively). Within the Hx-MBS subpopulation, the incidence of NASH and NAFLD was significantly higher among the obese compared to non-obese patients (1.84% vs 1.33%, *p* ≤ 0.001; 3.08% vs 1.86%, *p* ≤ 0.001, respectively), and, among NASH-Hx-MBS and NAFLD-Hx-MBS, there was no significant difference in the incidence of IBD for obese patients compared to non-obese patients (1.26% vs 1.39%, *p* = 0.577; 1.14% vs 1.13%, *p* = 0.930, respectively). With that said, among Hx-MBS, there was a higher incidence of NASH and NAFLD among those with IBD compared to those without IBD (2.69% vs 1.53%, *p* ≤ 0.001; 3.53% vs 2.33%, *p* ≤ 0.001, respectively), and significantly higher incidence of NASH and NAFLD among the obese compared to non-obese patients (3.76% vs 2.19%, *p* ≤ 0.001; 5.70% vs 2.50%, *p* ≤ 0.001, respectively). Over time, there was a positive temporal association between IBD, CMI, and hypothyroidism and Hx-MBS (*p* ≤ 0.001 for each) (Figures 2 and 3), and significantly higher odds of IBD for each year 2010–2020 compared to 2009 in the matched sample (*p* ≤ 0.001 for each).

Within the Hx-MBS subpopulation, liver disease, weight loss, blood loss anemia, or deficiency anemia was associated with higher odds of IBD (aOR:1.46, [1.27–1.68], *p* ≤ 0.001; aOR: 1.38, [1.21–1.57], *p* ≤ 0.001; aOR: 1.62, [1.29–2.05], *p* ≤ 0.001; aOR: 1.36, [1.21–1.53], *p* ≤ 0.001, respectively), but obesity was associated with lower odds of IBD (aOR: 0.81, [0.75–0.88], *p* ≤ 0.001) when controlling for other factors. Additionally, among Hx-MBS, deficiency anemia, vitamin B12 deficiency, or vitamin D deficiency was associated with significantly higher odds of *chronic thyroiditis* compared to others on univariate regression (OR:1.42, [1.23–1.65], *p* ≤ 0.001; OR: 2.26, [1.49–3.43], *p* ≤ 0.001; OR: 2.001, [1.64–2.45], *p* ≤ 0.001, respectively). Controlling for other factors, liver disease, cobalamin deficiency, vitamin D deficiency, obesity, or lymphoma was associated with significantly higher odds of hypothyroidism within the Hx-MBS subpopulation (aOR: 1.07, [1.04–1.10], *p* ≤ 0.001; aOR: 1.72, [1.08–1.27], *p* ≤ 0.001; aOR: 1.29, [1.24–1.33], *p* ≤ 0.001; aOR: 1.04, [1.03–1.06], *p* ≤ 0.001; aOR: 1.11, [1.01–1.22], *p* = 0.030). However, the odds of NASH were significantly higher for those with hypothyroidism and vitamin D deficiency (aOR: 1.89, [1.13–1.25], *p* ≤ 0.001; aOR: 1.57, [1.43–1.72], *p* ≤ 0.001), but significantly lower for those with cobalamin deficiency (aOR: 0.72, [0.57–0.97], *p* = 0.030). The odds of NAFLD were also significantly higher for those with hypothyroidism and vitamin D deficiency (aOR: 1.06 [1.01–1.10], *p* = 0.009; aOR: 1.68, [1.57–1.25], *p* ≤ 0.001). Peptic ulcer disease without bleeding was associated with increased odds of both NAFLD and NASH (aOR: 1.34 [1.24–1.45], *p* ≤ 0.001; aOR: 1.23, [1.11–1.36], *p* ≤ 0.001). Lastly, the odds of CMI were significantly higher for those with liver disease (aOR: 1.20, [1.02–1.40], *p* = 0.024; additional comorbid associations are noted in Online Supplement II and Supplementary Table 1-4).

Long-term aspirin (ASA) use was also associated with significantly lower odds of IBD within the unmatched and matched population and within an ASA-PSM Hx-MBS subpopulation (aOR: 0.80, [0.70–0.90], *p* = 0.002; aOR: 0.74, [0.64–0.86], *p* ≤ 0.001; aOR: 0.87, [0.78–0.99], *p* = 0.032, respectively); as well as lower odds of NAFLD and NASH within the unmatched regression (aOR: 0.87, [0.81–0.94], *p* ≤ 0.001; aOR: 0.81, [0.73–0.89], *p* ≤ 0.001).

On Hx-MBS-PSM analysis, Hx-MBS was associated with significantly higher odds of CMI, hypothyroidism, and IBD compared to matched controls within the PR-MBS population (aOR: 2.38, [2.08–2.74], *p* ≤ 0.001; aOR: 1.23, [1.21–1.26], *p* ≤ 0.001; aOR: 1.66, [1.52–1.82], *p* ≤ 0.001, respectively), but lower odds of NASH and NAFLD (aOR: 0.27, [0.25–0.28], *p* ≤ 0.001, aOR: 0.29, [0.28–0.30], *p* ≤ 0.001, respectively) (Table 2). Liver disease, weight loss, cobalamin, and vitamin D deficiency were associated with higher odds of IBD in the matched regression (aOR: 1.50, [1.37–1.63], *p* ≤ 0.001; aOR: 1.88, [1.74–2.02], *p* ≤ 0.001; aOR: 3.83, [3.14–4.69], *p* ≤ 0.001; aOR: 1.40, [1.23–1.60], *p* ≤ 0.001). Finally, even when controlling for the effects of CMI, NASH, and NAFLD which were each associated with higher odds of IBD comparatively (aOR: 2.74, [2.18–3.45], *p* ≤ 0.001; aOR: 1.56, [1.29–1.89], *p* ≤ 0.001; aOR: 1.27, [1.05–1.54], *p* = 0.012, respectively), Hx-MBS was still associated with significantly higher odds of IBD compared to matched PR-MBS controls (aOR: 1.48 [1.36–1.63], *p* ≤ 0.001).

**Table 2:** Hx-MBS and PR-MBS multivariate regression outcomes

	Unmatched (N: Hx-MBS-3,365,784; PR-MBS -1,050,900)	Matched (N: Hx-MBS- 626,041; PR-MBS - 198,846)
	Hx-MBS vs PR-MBS	Hx-MBS vs PR-MBS
IBD	1.56, [1.40–1.73], *p* ≤ 0.001	1.66, [1.52–1.82], *p* ≤ 0.001
CMI	3.52, [2.68–4.64], *p* ≤ 0.001	2.38, [2.08–2.74], *p* ≤ 0.001
NASH	0.31, [0.28–0.34], *p* ≤ 0.001	0.27, [0.25–0.28], *p* ≤ 0.001
NAFLD	0.26, [0.25–0.38], *p* ≤ 0.001	0.29, [0.28–0.30], *p* ≤ 0.001

## Discussion

### IBD and Metabolic Bariatric Surgery: Current and Past Research

In patients with severe obesity complicated by comorbidities, MBS has been an effective therapeutic measure against high adiposity, diabetes, and other sequelae associated with metabolic syndrome [1]. Our study highlights some of these health benefits and aligns well with previous research. However, our study demonstrates that among hospitalized patient encounters there were significantly higher odds of IBD, hypothyroidism, and CMI, and significantly lower odds of NASH and NAFLD for Hx-MBS compared to PR-MBS.

Patients with Hx-MBS are at increased risk of MD after surgery and decreased surface area for essential nutrient absorption within the digestive system [1–3]. Micronutrient malabsorption of cobalamin, vitamin D, etc., and their associated sequelae such as hypothyroidism, NAFLD, and NASH concur with known MD associations with these conditions, and the increased incidence of NAFLD/NASH among those with obesity [1–3, 21–37, 43–47, 54, 55]. Bariatric surgery may alter a key functional physiological step for cofactor conversion, binding, or absorption, indirectly altering gut symbiosis, increasing intestinal permeability, immune activation, and micro-nutritional status, or alternatively, exposing underlying genetic risk factors for development of IBD [1–3, 21–28, 43–45, 54, 55, 74, 75]. Moreover, other studies have postulated that alterations to enterohepatic bile acid circulation and associated changes in the GM play a critical role in metabolic alterations observed after MBS [12, 50, 51, 56–62, 76], but have also reported associated changes in hormonal regulation and mucosal crypt cell proliferation following Roux-en-Y gastric bypass (RYGB) [63–68]. The higher incidence of IBD among Hx-MBS patients with NAFLD or NASH compared to that of PR-MBS, and the significant association between Hx-MBS and IBD, controlling for the effects of cobalamin and vitamin D deficiency, suggests that the interplay between micronutrient absorption, gut dysbiosis, and immune or metabolic dysregulation associated with the MBS procedure may be implicated in the pathogenesis of IBD.

Our dataset does not allow us to determine which pathological conditions developed first and can only demonstrate significant associations between conditions in the described setting. As such, it is unclear if NASH and NAFLD among the Hx-MBS population developed prior to IBD presentation or after, and for how long disease conditions were present prior to the development of IBD. However, the lower odds of NASH/NAFLD among Hx-MBS compared to PR-MBS aligns well with recent research demonstrating lower incidence of major adverse liver outcomes for NAFLD patients receiving bariatric surgery compared to nonsurgical care [77]. Moreover, the significant association between NASH and NAFLD with IBD has been well established [24, 25, 78]. Sourianarayanane et al. noted significant associations between NAFLD and IBD and reported risk for NAFLD almost twice as high in patients with IBD compared to healthy subjects [25]. However, while previous studies have noted significantly lower incidence of NASH/NAFLD among those receiving anti-TNF alpha therapy [25], our study notes a protective effect against NASH, NAFLD, and IBD associated with ASA use among the bariatric surgery patient population.

Zamani et al. noted older, later age of onset of IBD, and fewer metabolic risk factors among the IBD patient population with NAFLD [37]. In our study, the mean age of IBD with or without NAFLD among the Hx-MBS population was significantly higher than PR-MBS, but significantly lower than those within the IBD subpopulation despite higher incidence compared to other IBD patients (see Supplement II). However, we did note a significant association with obesity and NAFLD among the Hx-MBS subpopulation. Additionally, our results demonstrate a significant association between cobalamin deficiency or vitamin D deficiency and IBD among the Hx-MBS inpatient cohort. The former aligns well with a recent meta-analysis utilizing a Mendelian randomization approach to characterize summary-level genome-wide association results from an international IBD consortia [29]. The study examined genetic variants and identified those strongly associated with measures of obesity and fat distribution, and blood levels of vitamins and fatty acids, and it demonstrated a significant positive association between variants associated with cobalamin deficiency and IBD, but did not note a significant association for variants of vitamin D deficiency [29].

Prior studies have also reported a significant difference in microflora biodiversity among IBD patients compared to others, noting major perturbations of the autochthonous bacterial flora (*Lactobacillus* and *Bifidobacterium* genera) and increased prevalence of anaerobic microbiota and facultative and conditionally pathogenic bacteria (*Bacteroides*, *Escherichia*, *Enterococcus*, and *Proteus*), and fewer *Clostridium leptum* and *Clostridium coccoides* subgroup populations and a decrease in the *Coprococcus* genera which contain many butyrate-producing microorganisms, believed to be involved in anti-inflammatory, immunomodulatory activity, and short-chain fatty acid production [53, 79–84]. Kushkevych et al. demonstrated in their study how sulfate-reducing bacteria (*Desulfovibrio*, *Desulfomicrobium*, *Lawsonia*, and *Bilophila* genera; species of the *Clostriudium* genus (SRB)), intestinal acetate accumulation which is also produced by SRB through incomplete oxidation of organic compounds, and greater concentration of intestinal hydrogen sulfide and associated increased intestinal permeability may be associated with UC [84]. Ulker et al., Ciobârcă et al., and Ilhan et al. characterize the significant differences in GM diversity between that of patients with Hx-MBS and others, and similarly, they note significantly higher relative number of species with sulfate-reducing bacteria, increased relative Bacteroidetes/Firmicutes ratio; alterations in prevalence of *E. coli*; decreases in butyrate-producing microorganisms; and more significant GM changes associated with RYGB [10, 11, 14, 19, 20].

### IBD and Metabolic Bariatric Surgery: Possible Mechanism and Targets for Future Research

A possible explanatory mechanism for these events could be related to low-grade inflammation and/or pro-mitotic reaction within the colonic mucosa. This pro-inflammatory, pro-mitotic pathophysiological state aligns well with prior research relating bile circulation and other pro-mitotic colonic exposures following MBS and reported increased mitotic activity among bile-exposed intestinal cells [63–68], and the pro-inflammatory state associated with other micronutrient deficiencies such as magnesium [68, 74–81]. The protective effects of aspirin noted in our study also support the role of an underlying pro-inflammatory, pro-mitotic state. Aspirin has both anti-inflammatory and potent immunomodulating effects, blunting immune cell and B-cell maturation and development, in addition to other immunomodulating activities [85]. Notably, a prior study reports that long-term aspirin use (LT-ASA) effectively decelerated epigenetic mitotic clock and increased intrinsic rate residuals—epigenetic aging measures that estimate cell division, where intrinsic rate (IR) = (mitotic clock/(chronological age at time point)), and IR residual = T2-T1—within the proximal colon for LT-ASA users compared to no LT-ASA at baseline (T1) and at 10-year follow-up (T2) [86]. The lower odds of IBD among those receiving aspirin may relate to this immunomodulatory effect.

Moreover, morbid/severe obesity (SO) itself has been associated with gut dysbiosis and inflammatory serum milieu [10, 12, 14, 43], and the resolution of this intrinsic inflammatory state with associated GM changes is believed to be associated with some of the benefits following MBS [10]. Notably, severe obesity was noted in prior analysis to have significant association with coagulopathy compared to others among the IBD subpopulation (Supplement II-Table 2-3) [87]. However, among the IBD-Hx-MBS subpopulation in our study, non-obese status, rheumatoid arthritis/collagen vascular disease, and coagulopathy were each significantly associated with IBD (see supplement II-Table 1). While an obesity-related inflammatory response may not be implicated in the pathology, GM dysbiosis and inflammation are not excluded from the disease etiology. Additionally, prior reports indicate that despite decreased Bacteroidetes/Firmicutes ratio, members of subdominant bacterial phylum Proteobacteria (gram-negative), which have greater endotoxic activity of the LPS molecule compared to dominant Bacteroidetes phylum, have been associated with obesity and insulin resistance, as well as a decreased prevalence of butyrate-producing Firmicutes among obese patients [18]. As such, further study examining how diet, obesity, GM, and associated factors impact IBD presentation requires further study.

The role compensatory mechanisms play in this pathophysiological process is unclear. Prior research has reported associations between SG and PUD [82, 83], and between proton pump inhibitor (PPI) use and magnesium and cobalamin deficiency [50, 51, 88]. In our study, PUD was associated with higher odds of NASH, NAFLD, and CMI (result not shown; See Supplement II). Factors such as comorbid PUD may be relevant secondary to treatment side effects, rather than or in addition to the primary disease pathology itself. For example, proton pump inhibitor use alters magnesium absorption within gastrointestinal endo/epithelium cells and increases the risk for hypomagnesemia—a critical micronutrient in thyroid function and essential for T4 to T3 conversion; and key factor in immune cell function, maturation, and development, activating phagocytic cells and initiating excessive production and release of (IL)-1β and tumor necrosis factor (TNF)-α; and also involved in platelet aggregation and adhesiveness and inhibition of endothelial growth and migration, potentially altering microvascular functions [69–73, 89–93].

Proton pump inhibitor use, in the setting of decreased total pepsin production secondary to reduced gastric volume in SG, may exacerbate increased luminal pH, which itself may affect bacterial growth [10, 12, 14, 88], and decrease intraluminal concentration of bound IF [88]; and alter reduction interactions of trivalent to absorbable, divalent cations (in SG) [12, 51, 55, 88]. Notably, we report a significant association between the presence of lymphoma and hypothyroidism; and chronic thyroiditis and vitamin B12, vitamin D, and deficiency anemia among Hx-MBS patients. As such, the combination of MD exacerbated by proton pump inhibitor (PPI) use in combination with baseline decreased pepsin and IF intraluminal concentrations (in SG) or proximal alimentary tract interactions (in RYGB); procedure-related divalent cation and micronutrient malabsorption, increased microbiome microbial diversity associated with relatively decreased luminal prevalence of butyrate-producing organisms and increased growth of sulfate-reducing bacteria within the gut; and increased exposure to pro-mitotic factors such as bile acid and undigested food compared to those without surgery, may represent a plausible mixture of factors for precipitation of IBD. However, the mechanism by which the interplay of micronutrients affects thyroid function has not been fully explained [55]; and the role that PUD and PPI therapy; biliary or gastric acid secretion; and diet plays in the modulation of these interactions requires further study.

Micronutrient deficiency such as hypomagnesemia, iron deficiency, vitamin B_12_ deficiency, vitamin D, and their associated clinical sequelae such as deficiency anemia, and the risk factors for developing these micronutrient deficiencies such as PPI use may represent valuable markers for increased risk (Supplement II-Table 1). In their study describing infliximab use in Crohn’s, Timmermans et al. describe signs of chronic B-cell stimulation with localization to granulomatous tissue and increased molecular maturation of IgA and IgG [17]. As such, serum immunoglobulin levels, antibody characterization, and immune regulatory mechanisms may represent focus areas to expand on our findings. Derangements within the structural features within the FAE or among DCs such as CD11c+CD11b+, as well as GM characterization may reveal additional factors associated with precipitation of IBD among Hx-MBS patients. These complex interactions effectuated through polygenic predisposition with multiple environmental interactions require additional research to understand how the GM, small bowel surgery, and liver disease interact in inflammatory bowel disease presentation [1–3, 21–37, 44].

### Limitations

Because we used the NIS to estimate outcomes, our findings can only be generalized to the inpatient setting, with associated results applicable to inpatient estimates only. The NIS identifies patient hospitalization encounters and recurrent hospitalizations may appear as distinct observations. Moreover, administrative coding variations and claim codes that do not affect reimbursement directly may be prone to variation in coding practices. As such, associated diagnosis among the respective populations is subject to coding variability, and the cases, behaviors, and clinical diagnoses using ICD codes are subject to errors in coding, and variability between coding conventions. However, the sample population adds power to the outcome analysis and represents a large, nationally representative inpatient sample dataset, incorporating a large multi-year study cohort population. Finally, the NIS does not provide information regarding causation of outcomes of interest, or other related outcomes, but correlation.

Due to the limitations associated with our dataset, we were not able to differentiate the type of bariatric surgery procedure performed among those in *Hx-MBS* within our study sample population—sleeve gastrectomy vs RYGB, vs other, nor the impact of diet; or time from procedural intervention to development of pathological conditions among those within Hx-MBS. Differences in the disease presentation may be related to the post-operative diet, type of procedure performed, and the duration since MBS was performed [1, 11, 12, 14]. However, similar micronutrient deficiencies have been reported in both SG and RYGB [4–9, 11, 12, 14], with prior studies suggesting that RYGB is associated with more severe micronutrient deficiencies [3, 11, 14, 15]. Notably, a recent study by Kiasat et al. reports that the RYGB and SG may individually be associated with increased risk for CD and UC, respectively [94].

## Conclusion

Because the bariatric surgery patient population represents one predisposed to chronic MD, they have increased relative susceptibility to long-term nutritional deficiencies and associated gut dysbiosis [3, 38, 95]. Screening guidelines have been presented to identify nutritional deficiencies among this population of patients, and prior studies have noted benefits associated with vitamin supplementation and higher incidence of MD among MBS patients non-adherent to vitamin supplementation [3, 19]. However, there has been marked difficulty in adherence to follow-up following procedural bariatric surgery with indications that SG follow-up data is worse than that for RYGB [79]. Inadequate follow-up was noted in a systemic review in JAMA in 2014 [80], and Switzer et al. noted that among a sample of 99 bariatric surgery studies only 40.4% of papers had adequate follow-up meeting McMaster criteria, while 42% had insufficient follow-up and 17.2% reported no follow-up [81]. Economic and structural barriers related to the cost of follow-up care, availability of appointments, and travel distance have previously been described, and the importance of follow-up care and attention to vitamin supplementation regimens with consideration for intramuscular injections should be emphasized for those patients who receive MBS [15, 79]. In our study, 60.68% of the hospitalized Hx-MBS population were not obese. As the MBS population ages, developing effective strategies to ensure adequate follow-up and increasing awareness among physicians are paramount.

Our study aligns with prior research examining a range of factors associated with IBD and demonstrates significant risk factors associated with IBD among the bariatric surgery population. These findings and the benefits associated with ASA use implicate an underlying chronic, inflammatory response associated with IBD among Hx-MBS patients. This inflammatory process may be associated with immune- and non-immune-mediated gastrointestinal pathophysiological adaptations. Additional studies examining outpatient healthcare expenditures and longer-term retrospective and prospective studies are needed to further validate these findings.

### Supplementary Information

ESM 1: (DOCX 1530 kb)

ESM 2: (PDF 163 kb)

## Data Availability

The datasets analyzed during the current study are available in the Healthcare Cost and Utilization Project (HCUP) repository, https://hcup-us.ahrq.gov/nisoverview.jsp.
