# Supplementary material for: Bariatric Surgery and Inflammatory Bowel Disease: National Trends and Outcomes Associated with Procedural Sleeve Gastrectomy vs Historical Bariatric Surgery Among US Hospitalized Patients 2009–2020
Source: Obes Surg. 2023 Oct 7;33(11):3472–86. doi: 10.1007/s11695-023-06833-7 (PMC10603008; doi:10.1007/s11695-023-06833-7)
Supplement: Supplementary file 1 — (DOCX 1530 kb) [file 11695_2023_6833_MOESM1_ESM.docx]

***Online Supplement I***

**ICD-9/ICD-10 CM Codes:**

***History of Bariatric Surgery:*** V4586, Z9884

***Inflammatory Bowel Disease (IBD):***

K5190, K5180, K5101, K5150, K5130, K5120, 5560, 5561, 5562, 5563, 5564, 5565, 5566, 5568, 5569, K5000, K50011, K50011, K50012, K50012, K50013, K50014, K50014, K50018, K50019, K5010, K50111, K50111, K50112, K50112, K50113, K50114, K50114, K50118, K50119, K5080, K50811, K50811, K50812, K50812, K50813, K50814, K50814, K50818, K50819, K5090, K50911, K50911, K50912, K50912, K50913, K50914, K50914, K50918, K50919, 5550, 5551, 5552, 5559

***Crohn's Disease:***

K5000, K50011, K50011, K50012, K50012, K50013, K50014, K50014, K50018, K50019, K5010, K50111, K50111, K50112, K50112, K50113, K50114, K50114, K50118, K50119, K5080, K50811, K50811, K50812, K50812, K50813, K50814, K50814, K50818, K50819, K5090, K50911, K50911, K50912, K50912, K50913, K50914, K50914, K50918, K50919, 5550, 5551, 5552, 5559

***Ulcerative Colitis:***

K5190, K5180, K5101, K5150, K5130, K5120, 5560, 5561, 5562, 5563, 5564, 5565, 5566, 5568, 5569

***Vitamin D Deficiency:***

2689, E559

***Vitamin B12 Deficiency:***

D511, D512, D513, D518, D519, D510, 2810, 2811

***Homocysteinemia:*** E7211, 2704

***Chronic Mesenteric Ischemia (CMI):***

K551, K558, K559, 5571, 5579

***NASH:***

5718, 5733, K7581

***NAFLD:***

5718, K760

***Hepatic Fibrosis/Sclerosis:*** 5719, K7400, K7401, K7402, K741, K742

***Chronic Thyroiditis:*** *2452, 2453, 2458, E063, E065*

***All-Use Cannabis:***

F1210, F1211, F12120, F12121, F12122, F12129, F1213, F12150, F12151, F12159, F12180, F12188, F1219, F1220, F1221, F12220, F12221, F12222, F12229, F1223, F12250, F12251, F12259, F1228, F12280, F12288, F1290, F1291, F12920, F12921, F12922, F12929, F1293, F12950, F12951, F12959, F12980, F12988, F1299

30520, 30521, 30522, 30523, 30430, 30431, 30432, 30433

***Cannabis Abuse:***

F1210, F1211, F12120, F12121, F12122, F12129, F1213, F12150, F12151, F12159, F12180, F12188, F1219, F1290, F1291, F12920, F12921, F12922, F12929, F1293, F12950, F12951, F12959, F12980, F12988, F1299

30430, 30431, 30432, 30433

***Cannabis Dependence:***

F1220, F1221, F12220, F12221, F12222, F12229, F1223, F12250, F12251, F12259, F1228, F12280, F12288, 30520, 30521, 30522, 30523

***Active Cannabis Use:***

F1210, F12120, F12121, F12122, F12129, F1213, F12150, F12151, F12159, F12180, F12188, F1219, F1290, F12920, F12921, F12922, F12929, F1293, F12950, F12951, F12959, F12980, F12988, F1299

30430, 30431, 30432

F1220, F12220, F12221, F12222, F12229, F1223, F12250, F12251, F12259, F1228, F12280, F12288

F1211, 30520, 30521, 30522

***Long-term (current) NSAID (Nonsteroidal Anti-inflammatory Drugs) Use:*** Z79.1

***Long-Term (current) Steroid Use:*** Z79.52

***Long-Term (current) Anticoagulation:*** Z79.01

***Long-term (current) Aspirin Use:*** Z79.82, V58.66

***Long-term (current) Drug Therapy (i.e., Remicade/ASA):*** Z79.899 Other long term (current) drug therapy

***Coronary Artery Disease/ Chronic ischemic heart disease:***

I25.10. I25.110, I25.111, I25.112, I25.118, I25.119

***Tobacco Use Disorder:***

F17203, F17208, F17209, F17210, F17211, F17213, F17218, F17219, F17220, F17221, F17223, F17228, F17290, F17291, F17293, F17298, F17299, F17.2, F17.203, F17.208, F17.209, F17.21, F17.210, F17.211, F17.213, F17.218, F17.219, F17.22, F17.220, F17.221, F17.223, F17.228, F17.29, F17.290, F17.291, F17.293, F17.298, F17.299

**Personal History IBD:** Z8719, V1279

**Non-infectious Colitis:** 5589, K529

**ICD-9/ ICD-10 PR Codes:**

***Bariatric Surgery:***

4382, 4389, 0DB64Z3, 0DB63ZZ

***Laparoscopic vertical (sleeve) gastrectomy:*** 4382, 0DB64Z3

***Open and other partial gastrectomy:*** 4389

***Excision of Stomach, Percutaneous Approach:*** 0DB63ZZ

***Roux-en-Y gastric bypass:*** 4438, 0D1647, 0D16474, 0D16479, 0D1647A, 0D1647B, 0D1647L, 0D164J4, 0D164J9, 0D164JA, 0D164JB, 0D164JL, 0D164K4, 0D164K9, 0D164KA, 0D164KB, 0D164KL, 0D164Z4, 0D164Z9, 0D164ZA, 0D164ZB, 0D164ZL

***Online Supplement II***

***Statistical Analysis***

**Definitions:**

The 2009 to 2020 National Inpatient Sample (NIS) databases were used to identify encounters with patients aged ≥18 years with procedure coding indicating MBS procedure (PR-MBS) during index admission according to International Classification of Diseases Ninth (ICD-9-PCS) or Tenth Revision (ICD-10-PCS) Procedure Coding System (Laparoscopic vertical (sleeve) gastrectomy ICD-9 PR: 4382, ICD-10 PR: 0DB64Z3; Open and other partial gastrectomy ICD-9 PR: 4389; Excision of Stomach, Percutaneous Approach: ICD-10 PR:0DB63ZZ). The sample included 5,440 procedural encounters for Roux-en-Y and 1,051,390 gastric sleeve procedural encounters.

**Metabolic and Bariatric Surgery:**

Procedural metabolic and bariatric surgery (PR-MBS) was defined as vertical sleeve gastrectomy (SG) for this study. However, alternative embodiments of bariatric surgery are currently in practice including adjustable gastric banding (AGB), duodenal switch (DS), Roux-en-Y gastric bypass (RYGB), biliopancreatic diversion (BPD), biliopancreatic diversion with duodenal switch (BPD-DS). The two most commonly performed procedures SG and RYGB are described below.^1^ Figures describing the different procedures and the recommended vitamin supplementation following MBS can be found in Figure 1 and Vitamin Supplementation table below.

- Sleeve gastrectomy is a restrictive procedure and involves excision of defined portions of the gastric tissue (~80% along the great curvature, including fundus, corpus, and antrum, with preservation of the pylorus) leaving a narrow “gastric sleeve” or reservoir with ~100mL capacity, and functions similarly to AGB by limiting the total food intake and promoting early satiety and decreasing ghrelin and other gastrointestinal (GI) hormones that reduce appetite. The procedure was initially carried out as a staged bariatric procedure, after which patients would convert VSG to RYGB or to BPD-DS, but the weight loss associated with the procedure alone was noted and outweighed additional risk of malabsorption associated with staged procedures. It should be noted that SG accelerates gastric emptying and intestinal motility and is associated with increased serum bile acids. Additionally, SG may worsen or trigger GERD symptoms in some individuals due to the increased pressure in the remaining stomach. The procedure is the most performed MBS procedure, and because it is not a malabsorption is believed to be associated with fewer micronutrient deficiencies than RYGB. However, it may be associated with decreased availability of pepsinogen and intrinsic factor within luminal fluid secondary to excision of gastric mucosa.
- RYGB is both a restrictive and malabsorptive procedure. Roux-en-Y gastric bypass involves the creation of a conduit circuit within the alimentary tract, circumventing transit through the distal stomach, duodenum, and proximal jejunum and directing food to an anastomotic canal at the jejuno-entero-junction. The procedure may or may not involve excision of portions of the stomach, however, the alimentary tract is not altered and secretions from the stomach and associated secretion-passage into the duodenum are maintained. The newly constructed gastric reservoir, separated from the remaining stomach, is anastomosed to the distal end of the transected small bowel, the Roux (alimentary) limb, and approximately 75–150 cm distal to the gastro-jejunostomy, the biliopancreatic limb is connected to the alimentary limb to create a common channel where absorption of food occurs. Thus, in addition to mechanical restriction of caloric intake, RYGB also impairs macro- and micro-nutrients’ absorption and facilitates increased bile acid exposure and absorption within the intestines. Dumping syndrome is more commonly associated with RYGB. It occurs when food moves too quickly from the stomach to the small intestine, leading to symptoms such as nausea, vomiting, diarrhea, dizziness, and sweating. Sleeve gastrectomy has a lower risk of dumping syndrome because the pylorus; the valve that controls stomach emptying, is preserved.^2^ In contrast to SG, RYGB has been shown to improve or resolve GERD (gastroesophageal reflux disease) symptoms in many cases.^3^ This procedure is currently the second most commonly performed MBS procedure with down trending procedural utilization.^1^ However, it is associated with worse micronutrient deficiency profile compared to SG.

**Figure 1: Common Bariatric Surgical Procedures**

.
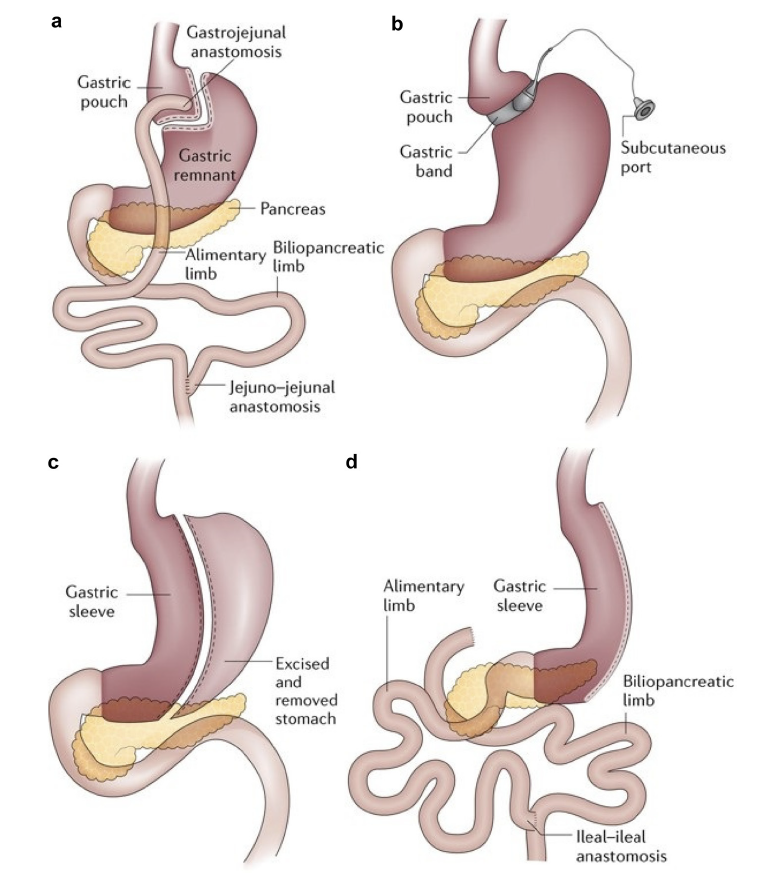


a: Roux-en-Y gastric bypass; b: adjustable gastric banding; c: sleeve gastrectomy; d: biliopancreatic diversion with duodenal switch.

Figure from [Common Bariatric Surgical Procedures](https://www.nature.com/articles/nrgastro.2016.170) by [Ninh T Nguyen](https://pubmed.ncbi.nlm.nih.gov/?term=Nguyen+NT&cauthor_id=27899816) et al licensed under Springer Nature^5^

**Micronutrient deficiencies following RYGB and VSG: Vitamin Supplementation**


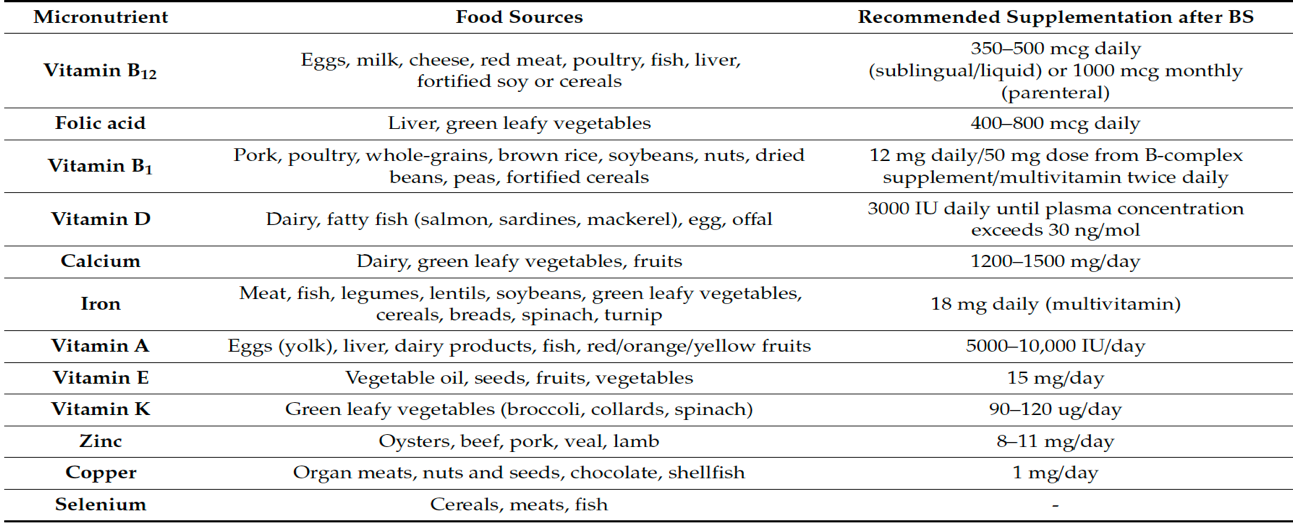


*RYGB—Roux-en-y gastric bypass; VSG—vertical sleeve gastrectomy.^7^*

*Table from* [*Micronutrient deficiencies following RYGB and VSG.*](https://www.ncbi.nlm.nih.gov/pmc/articles/PMC7019602/) *by* [Căto D. Ciobârcă](https://pubmed.ncbi.nlm.nih.gov/?term=Ciob%C3%A2rc%C4%83%20D%5BAuthor%5D) et al *under license* [*CC-BY*](https://creativecommons.org/licenses/by/4.0/)

**References**

1. *Estimate of bariatric surgery numbers, 2011-2020*. American Society for Metabolic and Bariatric Surgery. (2022, June 27). <https://asmbs.org/resources/estimate-of-bariatric-surgery-numbers>
2. Ramadan M, Loureiro M, Laughlan K, Caiazzo R, Iannelli A, Brunaud L, Czernichow S, Nedelcu M, Nocca D. Risk of Dumping Syndrome after Sleeve Gastrectomy and Roux-en-Y Gastric Bypass: Early Results of a Multicentre Prospective Study. Gastroenterol Res Pract. 2016; 2016:2570237. doi: 10.1155/2016/2570237. Epub 2016 May 8. PMID: 27242898; PMCID: PMC4875983.
3. Ralph Peterli, M. (2018, January 16). Effect of sleeve gastrectomy vs roux-en-Y gastric bypass on morbid obesity. JAMA.
4. Seeras K, Acho RJ, Lopez PP. Roux-en-Y Gastric Bypass Chronic Complications. [Updated 2022 Jun 11]. In: StatPearls [Internet]. Treasure Island (FL): StatPearls Publishing; 2023 Jan-. Available from: <https://www.ncbi.nlm.nih.gov/books/NBK519489/>
5. Nguyen NT, Varela JE. 2017. Bariatric surgery for obesity and metabolic disorders: state of the art. Nat Rev Gastroenterol Hepatol 14: 160–169.
6. Ulker İ, Yildiran H. The effects of bariatric surgery on gut microbiota in patients with obesity: a review of the literature. Biosci Microbiota Food Health. 2019;38(1):3-9. doi:10.12938/bmfh.18-018
7. Ciobârcă D, Cătoi AF, Copăescu C, Miere D, Crișan G. Bariatric Surgery in Obesity: Effects on Gut Microbiota and Micronutrient Status. Nutrients. 2020;12(1):235. Published 2020 Jan 16. doi:10.3390/nu12010235.

***Complex sampling design:***

Prior to 2012, the NIS was constructed annually by incorporating 100% of hospital discharges from 20% of U.S. hospitals. In 2012, the sampling design was reconstructed as a 20% national patient-level sample, with non-representative sampling across hospitals. These changes were associated with corresponding changes to sampling weights for patient encounters to estimate national outcomes. A new variable TEMPWT was created and equal to TRENDWT was utilized for discharges from 2009-2011; DISCWT was used for discharges from 2012-2020. A new variable HOSP_NISCOMBINE was created and equal to HOSPID was utilized for discharges from 2009-2011; HOSP_NIS was used for discharges from 2012-2020.

***Propensity Score Matching:***

The matching design used was 1:1 nearest neighbor with replacement using a 0.1 caliper width with common support. Variables were matched on age group, race, sex, admission year, hospital characteristics: hospital region, hospital bed size, location/teaching status of hospital; primary insurance payor: Medicare, Medicaid, Self-Pay, Other, No Charge; length of stay (LOS), total charge (TOTCHG), median household income for patient’s ZIP code, population median-Elixhauser-index sum score 3 or more, comorbidities, and long-term medications. STATA/MP software (StataCorp. 2021. Stata Statistical Software: Release 17. College Station, TX: StataCorp LLC) was used for all analyses.

***Supplementary Analysis, Tables, and Figures:***

1. **IBD Subpopulation and History of Bariatric Surgery:**

Using the NIS datasets from 2016 to 2020, we performed a sensitivity analysis for Hx-MBS outcomes, limiting to a cohort of hospital encounters age≥18 with ICD-10-CM coding indicating IBD diagnosis in any position of the medical claim. Within this subpopulation of hospitalized encounters with IBD admitted from 2016-2020 (N=1,423,626), there were 12,995 (0.91%) encounters associated with Hx-MBS with 83.10% females, p≤0.001, and mean age 53.66±0.27. Among those with no history of MBS (N-MBS_IBD) 56.02% were females, (p≤0.001), with mean age 54.27±0.04. The population median-Elixhauser sum was 3; mean was 2.986. The incidence of vitamin B12, vitamin D, and hypothyroidism were significantly higher for the Hx-MBS population compared to other IBD patients (0.92% vs 0.36%, p=≤0.001; 4.89% vs 3.02%, p=≤0.001; 20.78% vs 12.10%, p=≤0.001).

The incidence of NASH, NAFLD, and CMI was significantly higher for Hx-MBS compared to N-MBS_IBD (1.73% vs 0.54%, p=≤0.001; 3.50% vs 2.03%, p=≤0.001; 1.12% vs 0.78%, p=0.05). The mean age in years of those with NASH, NAFLD, or CMI was lower for those with Hx-MBS compared to others (55.58 vs 59.67, p=0.039; 50.42 vs 52.00, p=0.323; 58.28 vs 64.54, p=0.04, respectively). There was a lower incidence of HCC within the Hx-MBS population compared to others (0.42% vs 1.08%, p≤0.001, respectively), but no significant difference in mean age in years among patients with HCC for Hx-MBS compared to others (61.27 vs 61.15, p=0.978, respectively).

Hx-MBS encounters were matched to N-MBS_IBD encounters using propensity scores based on patient characteristics (Elixhauser comorbidities, history of tobacco use, coronary artery disease (CAD), median population Elixhauser sum, length of stay, total charge, admission on the weekend), hospital characteristics (region, location/teaching status, bed size), patient demographics (age, race, gender, income quartile based on ZIP code, primary insurance payor), and year of admission. In the PSM analysis, the odds of NASH or CMI were significantly higher for Hx-MBS compared to other IBD patients (aOR: 2.47, [1.548-3.953], p≤0.001; aOR: 1.77, [1.054-2.975], p=0.031, respectively); but not significantly different odds of NAFLD for Hx-MBS compared to N-MBS_IBD (aOR: 1.080, [0.838-1.393], p=0.552).

1. **IBD Subpopulation, Severe Obesity, and History of Bariatric Surgery:**

Inflammatory bowel disease (IBD) comprises Crohn's disease and ulcerative colitis. Morbid/Severe obesity (SO) is defined as body mass index (BMI) >40 kg/m2. Obesity is a known risk factor for developing IBD due to accumulation of intra-abdominal fat, cytokine production contributing to mucosal inflammation. Using the National Inpatient Sample (NIS), our sub-analysis examines trends and outcomes among IBD patients with and without SO.

The NIS database was used to identify patient encounters (pts) aged ≥18 years with diagnosis of IBD using ICD-10 codes between 2016-2019. Chi square, ANOVA, and multivariate regression were used to compare significant associations with variables and primary outcome of mortality among patients with IBD stratified by weight. Propensity Score Matching (PSM) was used to assess the effect of SO and history of Bariatric Surgery (BS) on the outcome of death. STATA MP 17 software was used for analysis.

Of the 1,111,015 patient encounters meeting inclusion criteria, 69,655 patients (69.61% female; mean age: 54 ±0.1424) had severe obesity. Among those without severe obesity 55.85% were female; with mean age 54±0.08 (p=0.227). There was a significant positive temporal relationship in death rate over time and severe obesity (p=0.001); but a significant negative relationship in death rate over time generally within the IBD population (p=0.015) (Supplementary Table 2; Figures A-C^1^). There was a positive trend in bariatric surgery over time within the general IBD population (p=0.0029) and among the IBD subpopulation with severe obesity (p≤0.001). Adjusted odds (aOR) of death were 43% higher among those with severe obesity compared to other weight groups (95%CI: 1.180-1.865, p≤0.001) (Figure A - C). Mean length of stay for those with severe obesity was 0.55 days longer than those without severe obesity–5.70 (95% CI:5.60- 5.80) Vs 5.17 (95% CI:5.10-5.20) days, respectively (p≤0.001). Results from the analysis also demonstrate 1) The odds of having bariatric surgery increases with increasing BMI, and patients with severe obesity have 46% higher odds of having had bariatric surgery compared to those without severe obesity in our study (p≤0.001) 2) There are 42% higher odds of having had bariatric surgery if the patient has had a colectomy compared to those without colectomy, and finally 3) The odds that a patient has had a colectomy are 12.8% lower in the severe obesity group compared to the others without severe obesity.

In propensity-score matched analysis, there was a 2.4% higher absolute risk of death for patients with severe obesity compared to those who did not have severe obesity (p= 0.030). There was a 0.698% lower absolute risk of death in patients with history of bariatric surgery compared to those without history of bariatric surgery within the general IBD population (p=0.026) and an 0.853% lower absolute risk of death when analysis was limited to the morbidly obese subpopulation (p=0.005) (Supplementary Table 3). While matched analysis allows for control of covariates, deeper, causal associations cannot be determined, and additional research is needed to further expand on these findings.

Among IBD pts, SO was associated with increased mortality compared to other weight classes (Figure A & B). Nonetheless, MBS within the entirety of the population was associated with decreased mortality. There may be some mortality benefit associated with higher weights-possibly related to medication side effects or markers of well controlled disease as weight loss itself was associated with increased odds of mortality (Figure A-C). More studies are warranted to analyze efficacy of MBS in other BMI groups.

**Reference:**

1. Surapaneni, Phani Keerthi MD; Igwe, Joseph-Kevin MD, MPH; Davisson, Laura MD; Beazer, Jabez K. MD;

Haggerty, Treah MD; Alabyad, Darwish MBBS; Mills, Krystal MD; Nguyen, Phuong MD; Adamson, Paula MD.

S976 *Recent Trends and Mortality Outcomes Among Inflammatory Bowel Disease Patients with and Without*

*Severe Obesity*. The American Journal of Gastroenterology 117(10S): p e707-e708, October 2022. | DOI:

10.14309/01.ajg.0000860544.28644.71

1. **Chronic Mesenteric Ischemia among those with History of Bariatric Surgery (2009-2020):**

Comorbid conditions significantly associated with Chronic Mesenteric Ischemia within the Hx-MBS subpopulation at p≤0.05 using the NIS 2009-2020:

**Higher Incidence:** Cardiac Arrhythmia (0.40% vs 0.31%, p≤0.001), Renal Failure (0.44% vs 0.32%, p≤0.001), PUD(0.43% vs 0.32%, p=0.010), Liver Disease (0.49% vs 0.32%, p≤0.001), Coagulopathy (0.66% vs 0.31%, p≤0.001), Weight Loss (0.82% vs 0.29%, p≤0.001), Fluid and Electrolyte Disorders (0.59% vs 0.23%, p≤0.001), Blood loss anemia (0.45% vs 0.33%, p=0.016), Complicated Hypertension (0.41% vs 0.32%, p≤0.001)

**Lower Incidence:** Pulmonary Circulation Disorder (0.24% vs 0.33%, p=0.010), Chronic Pulmonary Disease (0.29% vs 0.34%, p=0.002), Diabetes uncomplicated (0.29% vs 0.34%, p=0.005), Metastatic Cancer (0.18% vs 0.33%, p=0.013), Solid Tumor without metastasis (0.16% vs 0.33%, p≤0.001), Obesity (0.26% vs 0.37%, p≤0.001), Deficiency Anemia (0.26% vs 0.33%, p=0.002), Alcohol Abuse (0.23% vs 0.34%, p≤0.001), Psychosis (0.13% vs 0.33%, p≤0.001), Depression (0.31% vs 0.34%, p=0.035). The strong inhibitory effect on platelet aggregation and induction of CYP2C19 induced by antipsychotic medications such as clozapine, haloperidol, and risperidone may play a role in decreased risk of chronic mesenteric ischemia associated with history of psychosis.^1^

Reference:

1. Wu CC, Tsai FM, Chen ML, et al. Antipsychotic Drugs Inhibit Platelet Aggregation via P2Y1 and P2Y12 Receptors. Biomed Res Int. 2016; 2016: 2532371. doi:10.1155/2016/2532371

**Supplementary Table 1: Hx-MBS-IBD Subpopulation (2009-2020)**

|  | **IBD** | | **No IBD** | |
| --- | --- | --- | --- | --- |
| **FREQUENCY (Encounters)** | 25,401 | | 3,340,383 | |
| **ELIXHAUSER COMORBIDITIES** | **Mean** | **Linearized Standard error (s.e.)** | **Mean** | **Linearized Standard error (s.e.)** |
| **Age in years at admission** | 52.08242** | 0.1963449 | 53.51448** | 0.0387029 |
| **Sex** |  |  |  |  |
| Male | 0.1678057** | 0.0058064 | 0.213225 ** | 0.0006739 |
| Female | 0.8318705** | 0.0058091 | 0.7868944** | 0.0006742 |
| Race |  |  |  |  |
| White | 0.8245831** | 0.0059386 | 0.7466715** | 0.0018678 |
| Black | 0.0986205** | 0.004657 | 0.1394053** | 0.0013131 |
| Hispanic | 0.0545604** | 0.0034693 | 0.0818391** | 0.0012362 |
| Asian/Pacific Islander | 0.001853** | 0.0006233 | 0.004796** | 0.0001885 |
| Native American | 0.0030883 | 0.0007965 | 0.0047598** | 0.0002065 |
| Other | 0.0172946** | 0.0019916 | 0.0225283** | 0.000579 |
| **Comorbid Conditions** |  |  |  |  |
| **Congestive Heart Failure** | 0.0752467** | 0.0039202 | 0.1075233** | 0.3097776 |
| **Cardiac Arrhythmias** | 0.141383** | 0.0050622 | 0.1710809** | 0.0007109 |
| **Valvular Disease** | 0.0346153** | 0.0026867 | 0.0414795** | 0.0003277 |
| **Pulmonary Circulation Disorders** | 0.0327851 | 0.0026269 | 0.0374418 | 0.0002899 |
| **Peripheral Vascular Disorders** | .036965 | 0.0028008 | 0.0400498 | 0.0003416 |
| **Hypertension Uncomplicated** | 0.2175862** | 0.0060868 | 0.279526** | 0.0014701 |
| **Paralysis** | 0.0048768** | 0.0010101 | 0.0101247** | 0.0001374 |
| **Other Neurological Disorders** | 0.1024807** | 0.0044892 | 0.0823542** | 0.0004525 |
| **Chronic Pulmonary Disease** | 0.2553871** | 0.0064895 | 0.2279907** | 0.0007618 |
| **Diabetes Uncomplicated** | 0.1484939** | 0.0052369 | 0.1765221** | 0.0006452 |
| **Diabetes Complicated** | 0.0803116** | 0.0040445 | 0.0968825** | 0 .00072 |
| **Hypothyroidism** | 0.2005262** | 0.0060661 | 0.182737** | 0.0006454 |
| **Renal Failure** | 0.0912487** | 0.0043104 | 0.1030682** | 0.0006326 |
| **Liver Disease** | 0.0883429** | 0.0042035 | 0.0673726** | 0.0004297 |
| **PUD WO Bleeding** | 0.0401104** | 0.0028388 | 0.0316451** | 0.0003397 |
| **Lymphoma** | 0.0067685 | 0.0012402 | 0.0059721 | 0.000121 |
| **Metastatic Cancer** | 0.0079113** | 0.0013207 | 0.0144117** | 0.0002031 |
| **Solid Tumor Without Metastasis** | 0.0196983** | 0.0020287 | 0.0307367** | 0.0003078 |
| **Rheumatoid Arthritis/ Collagen Vascular** | 0.082696** | 0.0041286 | 0.0430207** | 0.0002902 |
| **Coagulopathy** | 0.0594079** | 0.0033612 | 0.0509221** | 0.0003775 |
| **Obesity** | 0.3222512** | 0.0069026 | 0.3937407** | 0.0012458 |
| **Weight Loss** | 0.1150022** | 0.0049493 | 0.0644694** | 0.0005113 |
| **Fluid and Electrolyte Disorders** | 0.3411188** | 0.0069276 | 0.260323** | 0.0009065 |
| **Blood Loss Anemia** | 0.0268143** | 0.00228 | 0.0166817** | 0.000184 |
| **Deficiency Anemia** | 0.1283019** | 0.0047728 | 0.0958519** | 0.0004924 |
| **Alcohol Abuse** | 0.0533479** | 0.0032959 | 0.0802118** | 0.0005845 |
| **Drug Abuse** | 0.0823087** | 0.0040885 | 0.0618974** | 0.0004856 |
| **Psychoses** | 0.0151084 | 0.0018622 | 0.0164412 | 0.0001956 |
| **Depression** | 0.3333683** | 0.0070335 | 0.2988331** | 0.0010345 |
| **Complicated Hypertension** | 0.0988642** | 0.0044497 | 0.121726** | 0.0008327 |
| **Aspirin** | 0 .057857** | 0.0033646 | 0.0805781** | .0007163 |
| **Morbid Obesity** | 0.1911643** | 0.0057993 | 0.254180** | 0.0009903 |
| **Vitamin B12 Deficiency** | 0.0087121*** | 0.0013792 | 0.0066693*** | 0.0001171 |
| **Vitamin D Deficiency** | 0.038564** | 0.0027495 | 0.0333118** | 0.0003672 |
| **Elixhauser Sum≥ 4** | 0.2592426 | 0.0070524 | 0.2515699 | 0.0024421 |
| ****: P less than or equal to .05 on χ2 analysis**  *****: P=0.067** | | | | |

**Supplementary Table 2: IBD Subpopulation 2016-2019**

| **Demographics Results** | | | | | |
| --- | --- | --- | --- | --- | --- |
|  | **SEVERE OBESITY** | |  | **SEVERE OBESITY** | |
|  | **YES** | **NO** |  | **YES** | **NO** |
| **FREQUENCY** | 69,655 | 1,111,015 |  | | |
| **MEAN (SD)** | | | **TOTAL CHARGE CATEGORICAL GROUPED** | | |
| **Age in years at admission (SD)** | 54.54**(0.14) | 54.05** (.08027) | **50000-99,999** | 64.17%** | 68.72%** |
| **Length of stay (SD)** | 5.701** (0.061) | 5.165** (0.02) | **100k-249,999** | 21.79%** | 19.53%** |
| **Total charges (SD)** | 61678.96**(936.39) | 54495.4** (492.93) | **250k-499,999** | 11.28%** | 9.56%** |
|  | | | **250k-499,999** | 2.24%** | 1.76%** |
| **FACTOR VARIABLE MEAN PERCENT** | | | **500k-1m** | 0.52%** | 0.43%** |
| **Died during hospitalization** | 1.40% | 1.40% | **LOCATION & TEACHING STATUS OF HOSPITAL** | | |
| **AGE GROUPS, RACE, & SEX** | | | **Rural** | 8.00%** | 7.35%** |
| **AGE 18-29** | 5.54% ** | 12.95% ** | **Urban Non-Teaching** | 21.13%** | 19.89%** |
| **AGE 30-54** | 41.87% ** | 36.16% ** | **Urban Teaching** | 70.86%** | 72.76%** |
|  |  |  | **Northeast** | 18.54%** | 21.72%** |
| **AGE 55-64** | 23.39% ** | 16.93% ** | **Midwest** | 29.93%** | 24.80%** |
| **AGE 65-119** | 29.20% ** | 33.96% ** | **South** | 36.53%** | 36.56%** |
| **WHITE** | 79.69% ** | 79.65% ** | **West** | 15.00%** | 16.93%** |
|  |  |  | **FACTOR VARIABLE MEAN PERCENT** | | |
| **BLACK** | 13.05% ** | 11.04% ** | **Congestive Heart Failure** | 21.99** | 10.83%** |
| **HISPANIC** | 4.64% ** | 5.60% ** |  |  |  |
| **ASIAN/PACIFIC ISLANDER** | 0.32% ** | 1.14% ** | **Cardiac Arrhythmias** | 22.19%** | 17.60%** |
| **NATIVE AMERICAN** | 0.61% ** | 0.35%** | **Valvular Disease** | 4.92% | 4.75% |
| **OTHER RACE** | 1.68% ** | 2.22%** | **Pulmonary Circulation Disorders** | 7.44%** | 3.37%** |
| **MALE** | 30.39% ** | 44.15%** |  |  |  |
| **FEMALE** | 69.61%** | 55.85%** | **Peripheral Vascular Disorders** | 5.86% | 5.67% |
| **PRIMARY EXPECTED PAYER** | | | **Other Neurological Disorders** | 9.20% | 8.97% |
| **Medicare** | 46.65%** | 43.13%** | **Uncomplicated Diabetes** | 16.90%** | 7.96%** |
|  |  |  | **Complicated Diabetes** | 25.07%** | 8.88%** |
| **Medicaid** | 15.04%** | 14.58%** | **Liver Disease** | 9.48%** | 6.84%** |
| **Private Insurance** | 33.52%** | 35.91%** | **Metastatic Cancer** | 1.41%** | 2.30%** |
| **Self-pay** | 2.51%** | 3.39%** | **Coagulopathy** | 7.54%** | 6.61%** |
| **No charge** | 0.26%** | 0.34%** | **Weight Loss** | 5.11%** | 13.21%** |
| **Other** | 2.03%** | 2.64%** | **Fluid & Electrolyte Disorders** | 35.48%** | 37.39%** |
| **LENGTH OF STAY** | | |  |  |  |
| **Less than 30 days** | 98.83%** | 99.05%** | **Iron Deficiency Anemia** | 7.98% | 8.36% |
| **30-59 days** | 0.98% | 0.81%** | **Drug Abuse** | 5.35%** | 7.33%** |
|  |  |  | **Depression** | 25.73%** | 18.79%** |
| **60-119 days** | 0.18%** | 0.11%** | **Long-term Steroid Use** | 5.69%** | 6.17%** |
|  |  |  | **Long-term Remicade/ASA** | 15.72%** | 14.74%** |
| **120-364** | 0.01%** | 0.02%** | **Long-term Anticoagulation** | 10.91%** | 7.19%** |
| **BED SIZE OF HOSPITAL** | | | **History of Colectomy** | 14.61%** | 16.34%** |
| **Small** | 20.21%** | 19.57%** | **History of Bariatric Surgery** | 3.04%** | 0.78%** |
|  |  |  | **TABLE LEGEND:** | | |
| **Medium** | 28.40%** | 27.98%** | ****: P less than or equal to 0.05** | | |
| **Large** | 51.39%** | 52.45%** |  |  |  |

**Supplementary Table 3: IBD Subpopulation—Severe Obesity vs Others**

| **Matched Sample Analysis: IBD Population** | | |
| --- | --- | --- |
| **SEVERE OBESITY** | **YES (CI)** | **NO (CI)** |
| **FREQUENCY** | **13,360** | **21,350** |
| **MEAN % DIED** | 1.37% (.0108- .0139) ** | 1.23% (.0118 - .0158) ** |
| **Mean (Standard Deviation (SD))** |  |  |
| **Age in years at admission** | 54.52(.14) ** | 55.59 (0.14) ** |
| **Mean Length of stay in Days (SD)** | 5.71 (.061) ** | 6.28(.05) ** |
| **Mean Total charges in US Dollars (SD)** | $61,970.03 (978.97) ** | $64,792.88 (805.86) ** |
| **Matched Sample Analysis: IBD Population** | | |
| **History of Bariatric Surgery** | **Yes (CI)** | **No (CI)** |
| **FREQUENCY** | **2,032** | **220,171** |
| **MEAN % DIED** | 0.69%** | 1.40%** |
| **Age in years at admission** | 53.39 (0.30) ** | 54.17 (0.08) ** |
| **Mean Length of stay in Days (SD)** | 4.87 (.12) ** | 5.20 (0.02) ** |
| **Mean Total charges in US Dollars (SD)** | $54,250.14 (1,670.12) ** | $55,050.96 (487.44) ** |
| **TABLE LEGEND:** | | |
| ****: P less than or equal to 0.05** | | |

**Figure A: IBD Subpopulation Mortality Rate over Weight Group**


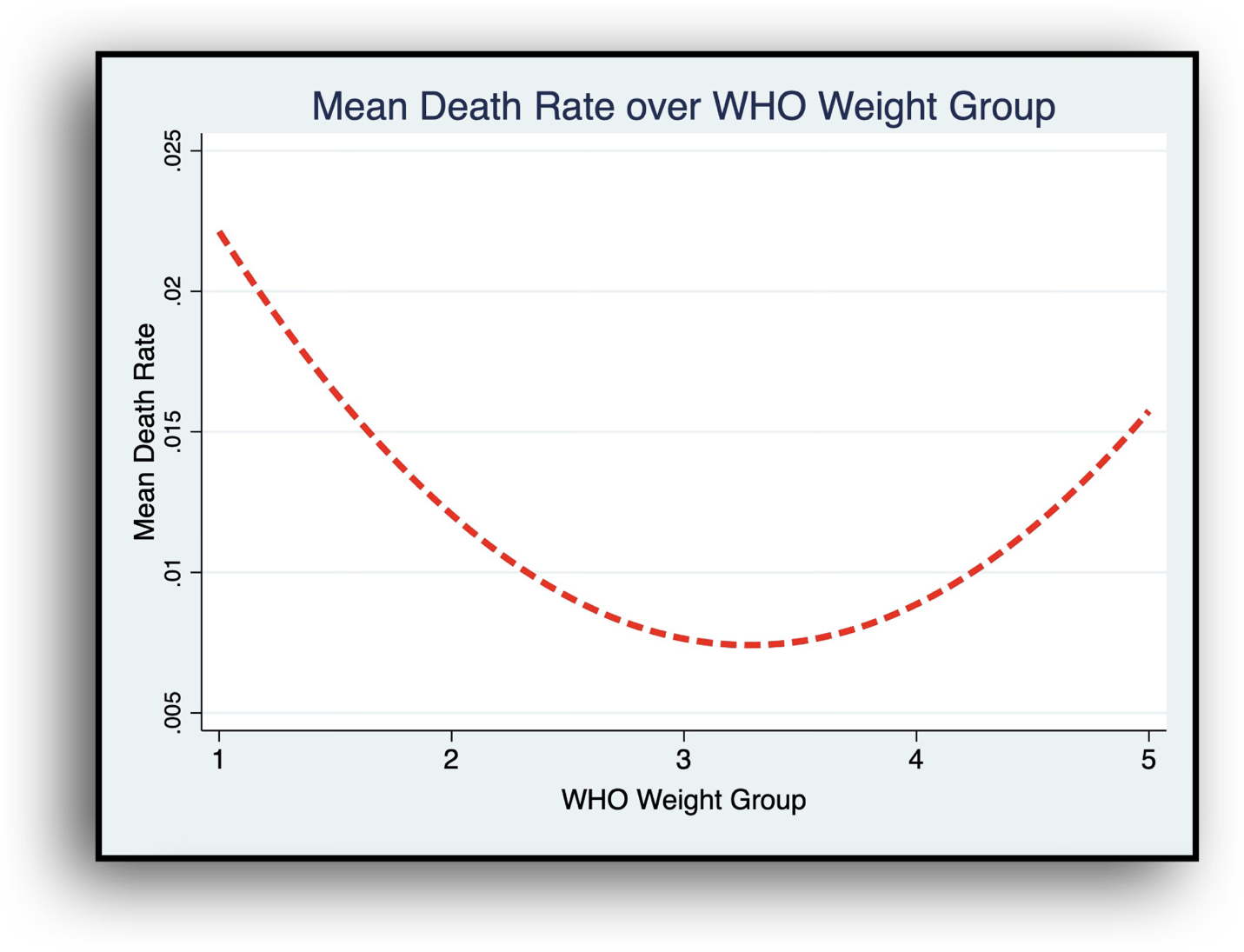


**Figure B: IBD Subpopulation Mortality Rate over Time by Weight Group**


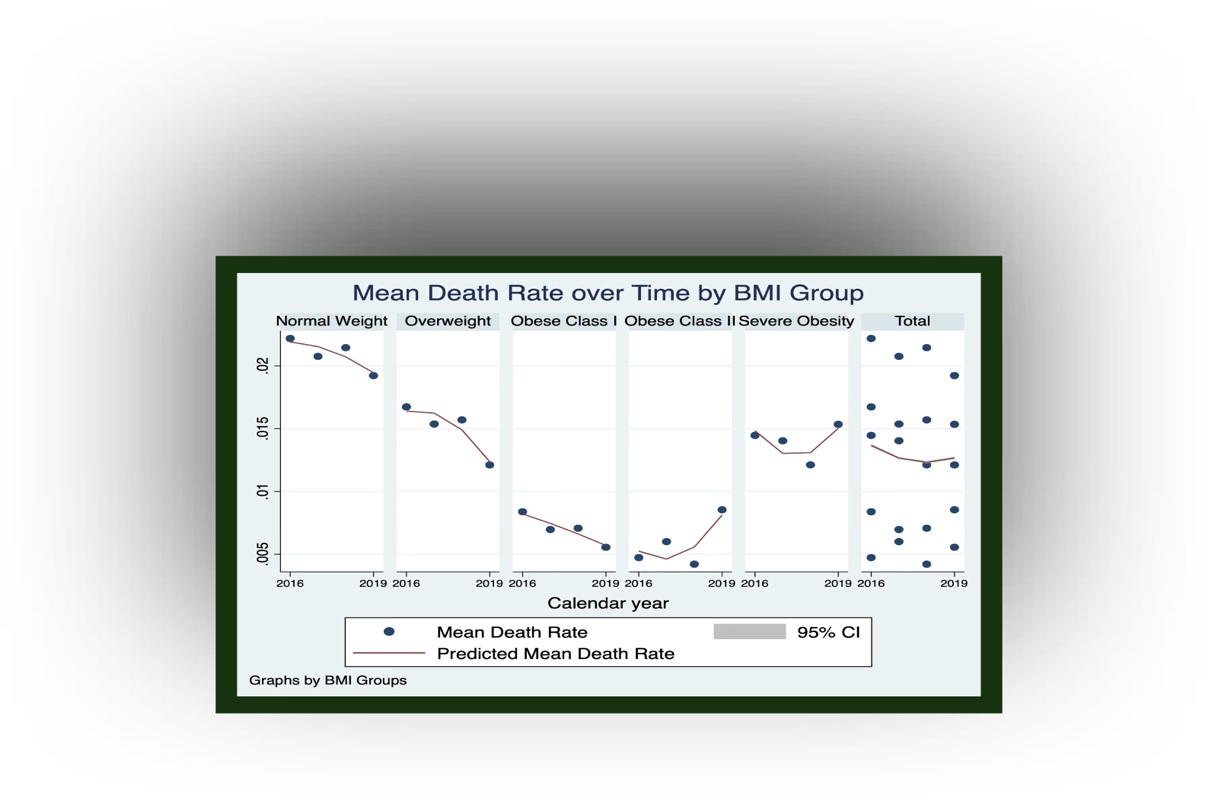


**Figure C: IBD Subpopulation Incidence of Non-Infectious Colitis by Weight Group**


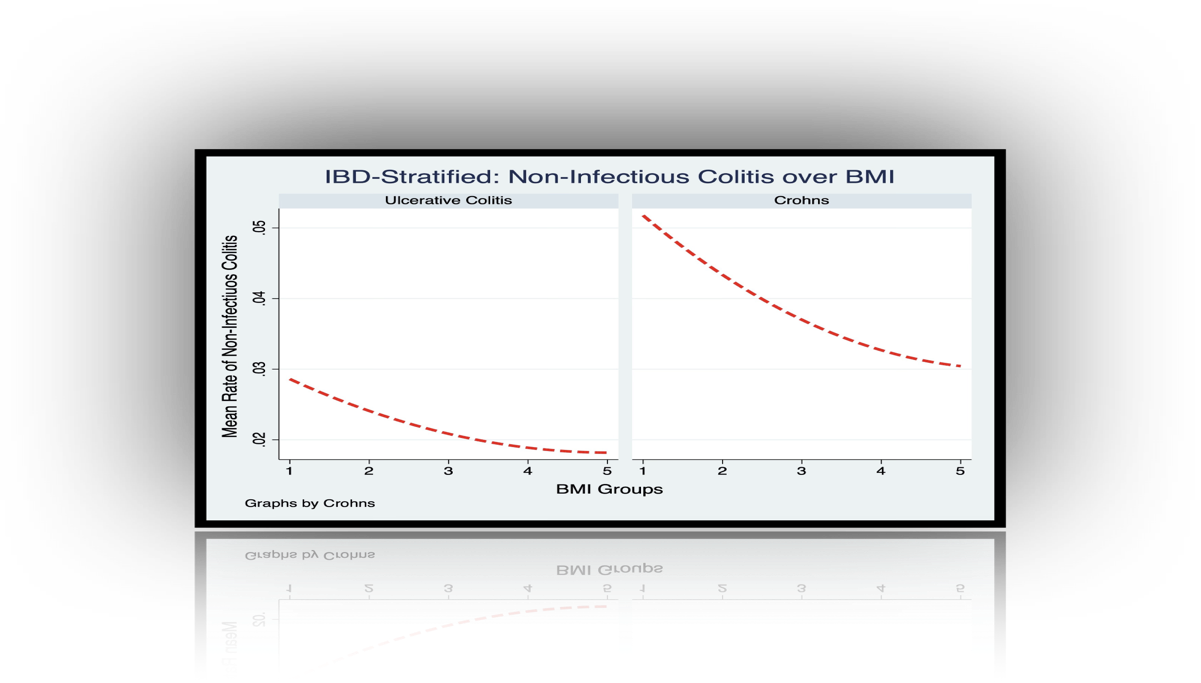


**Reference (Figure A-C):**

Surapaneni, Phani Keerthi MD; Igwe, Joseph-Kevin MD, MPH; Davisson, Laura MD; Beazer, Jabez K. MD;

Haggerty, Treah MD; Alabyad, Darwish MBBS; Mills, Krystal MD; Nguyen, Phuong MD; Adamson, Paula MD.

S976 *Recent Trends and Mortality Outcomes Among Inflammatory Bowel Disease Patients with and Without*

*Severe Obesity*. The American Journal of Gastroenterology 117(10S): p e707-e708, October 2022. | DOI:

10.14309/01.ajg.0000860544.28644.71
