# Supplementary material for: Bariatric Surgery and Inflammatory Bowel Disease: National Trends and Outcomes Associated with Procedural Sleeve Gastrectomy vs Historical Bariatric Surgery Among US Hospitalized Patients 2009–2020
Source: Obes Surg. 2023 Oct 7;33(11):3472–86. doi: 10.1007/s11695-023-06833-7 (PMC10603008; doi:10.1007/s11695-023-06833-7)
Supplement: Supplementary file 2 — (PDF 163 kb) [file 11695_2023_6833_MOESM2_ESM.pdf]

SPRINGER NATURE LICENSE
TERMS AND CONDITIONS

Sep 03, 2023

This Agreement between Dr. Joseph-Kevin Igwe ("You") and Springer Nature ("Springer Nature") consists of your license details and the terms and conditions provided by Springer Nature and Copyright Clearance Center.

License Number	5621561032686
License date	Sep 03, 2023
Licensed Content Publisher	Springer Nature
Licensed Content Publication	Nature Reviews Gastroenterology & Hepatology
Licensed Content Title	Bariatric surgery for obesity and metabolic disorders: state of the art
Licensed Content Author	Ninh T. Nguyen et al
Licensed Content Date	Nov 30, 2016
Type of Use	Journal/Magazine
Requestor type	publisher
Publisher	Springer Nature
Is this reuse sponsored by or associated with a pharmaceutical or a medical products company?	no
Format	print and electronic

Portion	figures/tables/illustrations
Number of figures/tables/illustrations	1
Will you be translating?	no
Publishing Open Access	Yes
Creative Commons License	CC-BY
Circulation/distribution	20000 - 49999
Author of this Springer Nature content	no
Title of new article	Bariatric Surgery and Inflammatory Bowel Disease: National Trends and Outcomes Associated with Procedural Sleeve Gastrectomy vs Historical Bariatric Surgery among US Hospitalized Patients 2009-2020
Lead author	Joseph-Kevin Igwe
Title of targeted journal	Obesity Surgery The Journal of Metabolic Surgery and Allied Care
Publisher	Springer Nature
Expected publication date	Oct 2023
Order reference number	BARIATRICSURGERYTYPE
Portions	Figure 1: Common bariatric surgical procedures
Requestor Location	Dr. Joseph-Kevin Igwe<br>15210 Newport Bridge Circle<br><br>SUGAR LAND, TX 77498

	United States<br>Attn: MPH
Total	0.00 USD

Terms and Conditions

Springer Nature Customer Service Centre GmbH Terms and Conditions

The following terms and conditions ("Terms and Conditions") together with the terms specified in your [RightsLink] constitute the License ("License") between you as Licensee and Springer Nature Customer Service Centre GmbH as Licensor. By clicking 'accept' and completing the transaction for your use of the material ("Licensed Material"), you confirm your acceptance of and obligation to be bound by these Terms and Conditions.

1. Grant and Scope of License

1. 1. The Licensor grants you a personal, non-exclusive, non-transferable, non-sublicensable, revocable, world-wide License to reproduce, distribute, communicate to the public, make available, broadcast, electronically transmit or create derivative works using the Licensed Material for the purpose(s) specified in your RightsLink Licence Details only. Licenses are granted for the specific use requested in the order and for no other use, subject to these Terms and Conditions. You acknowledge and agree that the rights granted to you under this License do not include the right to modify, edit, translate, include in collective works, or create derivative works of the Licensed Material in whole or in part unless expressly stated in your RightsLink Licence Details. You may use the Licensed Material only as permitted under this Agreement and will not reproduce, distribute, display, perform, or otherwise use or exploit any Licensed Material in any way, in whole or in part, except as expressly permitted by this License.

1. 2. You may only use the Licensed Content in the manner and to the extent permitted by these Terms and Conditions, by your RightsLink Licence Details and by any applicable laws.

1. 3. A separate license may be required for any additional use of the Licensed Material, e.g. where a license has been purchased for print use only, separate permission must be obtained for electronic re-use. Similarly, a License is only valid in the language selected and does not apply for editions in other languages unless additional translation rights have been granted separately in the License.

1. 4. Any content within the Licensed Material that is owned by third parties is expressly excluded from the License.

1. 5. Rights for additional reuses such as custom editions, computer/mobile applications, film or TV reuses and/or any other derivative rights requests require additional permission and may be subject to an additional fee. Please apply to journalpermissions@springernature.com or bookpermissions@springernature.com for these rights.

2. Reservation of Rights

Licensor reserves all rights not expressly granted to you under this License. You acknowledge and agree that nothing in this License limits or restricts Licensor's rights in or use of the Licensed Material in any way. Neither this License, nor any act, omission, or statement by Licensor or you, conveys any ownership right to you in any Licensed Material, or to any element or portion thereof. As between Licensor and you, Licensor owns and retains all right, title, and interest in and to the Licensed Material subject to the license granted in Section 1.1. Your permission to use the Licensed Material is expressly conditioned on you not impairing Licensor's or the applicable copyright owner's rights in the Licensed Material in any way.

3. Restrictions on use

3. 1. Minor editing privileges are allowed for adaptations for stylistic purposes or formatting purposes provided such alterations do not alter the original meaning or intention of the Licensed Material and the new figure(s) are still accurate and representative of the Licensed Material. Any other changes including but not limited to, cropping, adapting, and/or omitting material that affect the meaning, intention or moral rights of the author(s) are strictly prohibited.

3. 2. You must not use any Licensed Material as part of any design or trademark.

3. 3. Licensed Material may be used in Open Access Publications (OAP), but any such reuse must include a clear acknowledgment of this permission visible at the same time as the figures/tables/illustration or abstract and which must indicate that the Licensed Material is not part of the governing OA license but has been reproduced with permission. This may be indicated according to any standard referencing system but must include at a minimum 'Book/Journal title, Author, Journal Name (if applicable), Volume (if applicable), Publisher, Year, reproduced with permission from SNCSC'.

4. STM Permission Guidelines

4. 1. An alternative scope of license may apply to signatories of the STM Permissions Guidelines ("STM PG") as amended from time to time and made available at https://www.stm-assoc.org/intellectual-property/permissions/permissions-guidelines/.

4. 2. For content reuse requests that qualify for permission under the STM PG, and which may be updated from time to time, the STM PG supersede the terms and conditions contained in this License.

4. 3. If a License has been granted under the STM PG, but the STM PG no longer apply at the time of publication, further permission must be sought from the Rightsholder. Contact journalpermissions@springernature.com or bookpermissions@springernature.com for these rights.

5. Duration of License

5. 1. Unless otherwise indicated on your License, a License is valid from the date of purchase ("License Date") until the end of the relevant period in the below table:

Reuse in a medical communications project	Reuse up to distribution or time period indicated in License

Reuse in a dissertation/thesis	Lifetime of thesis
Reuse in a journal/magazine	Lifetime of journal/magazine
Reuse in a book/textbook	Lifetime of edition
Reuse on a website	1 year unless otherwise specified in the License
Reuse in a presentation/slide kit/poster	Lifetime of presentation/slide kit/poster. Note: publication whether electronic or in print of presentation/slide kit/poster may require further permission.
Reuse in conference proceedings	Lifetime of conference proceedings
Reuse in an annual report	Lifetime of annual report
Reuse in training/CME materials	Reuse up to distribution or time period indicated in License
Reuse in newsmedia	Lifetime of newsmedia
Reuse in coursepack/classroom materials	Reuse up to distribution and/or time period indicated in license

6. Acknowledgement

6. 1. The Licensor's permission must be acknowledged next to the Licensed Material in print. In electronic form, this acknowledgement must be visible at the same time as the figures/tables/illustrations or abstract and must be hyperlinked to the journal/book's homepage.

6. 2. Acknowledgement may be provided according to any standard referencing system and at a minimum should include "Author, Article/Book Title, Journal name/Book imprint, volume, page number, year, Springer Nature".

7. Reuse in a dissertation or thesis

7. 1. Where 'reuse in a dissertation/thesis' has been selected, the following terms apply: Print rights of the Version of Record are provided for; electronic rights for use only on institutional repository as defined by the Sherpa guideline (www.sherpa.ac.uk/romeo/) and only up to what is required by the awarding institution.

7. 2. For theses published under an ISBN or ISSN, separate permission is required. Please contact journalpermissions@springernature.com or bookpermissions@springernature.com for these rights.

7. 3. Authors must properly cite the published manuscript in their thesis according to current citation standards and include the following acknowledgement: '*Reproduced with permission from Springer Nature*'.

8. License Fee

You must pay the fee set forth in the License Agreement (the "License Fees"). All amounts payable by you under this License are exclusive of any sales, use, withholding, value added or similar taxes, government fees or levies or other assessments. Collection

and/or remittance of such taxes to the relevant tax authority shall be the responsibility of the party who has the legal obligation to do so.

9. Warranty

9. 1. The Licensor warrants that it has, to the best of its knowledge, the rights to license reuse of the Licensed Material. **You are solely responsible for ensuring that the material you wish to license is original to the Licensor and does not carry the copyright of another entity or third party (as credited in the published version).** If the credit line on any part of the Licensed Material indicates that it was reprinted or adapted with permission from another source, then you should seek additional permission from that source to reuse the material.

9. 2. EXCEPT FOR THE EXPRESS WARRANTY STATED HEREIN AND TO THE EXTENT PERMITTED BY APPLICABLE LAW, LICENSOR PROVIDES THE LICENSED MATERIAL "AS IS" AND MAKES NO OTHER REPRESENTATION OR WARRANTY. LICENSOR EXPRESSLY DISCLAIMS ANY LIABILITY FOR ANY CLAIM ARISING FROM OR OUT OF THE CONTENT, INCLUDING BUT NOT LIMITED TO ANY ERRORS, INACCURACIES, OMISSIONS, OR DEFECTS CONTAINED THEREIN, AND ANY IMPLIED OR EXPRESS WARRANTY AS TO MERCHANTABILITY OR FITNESS FOR A PARTICULAR PURPOSE. IN NO EVENT SHALL LICENSOR BE LIABLE TO YOU OR ANY OTHER PARTY OR ANY OTHER PERSON OR FOR ANY SPECIAL, CONSEQUENTIAL, INCIDENTAL, INDIRECT, PUNITIVE, OR EXEMPLARY DAMAGES, HOWEVER CAUSED, ARISING OUT OF OR IN CONNECTION WITH THE DOWNLOADING, VIEWING OR USE OF THE LICENSED MATERIAL REGARDLESS OF THE FORM OF ACTION, WHETHER FOR BREACH OF CONTRACT, BREACH OF WARRANTY, TORT, NEGLIGENCE, INFRINGEMENT OR OTHERWISE (INCLUDING, WITHOUT LIMITATION, DAMAGES BASED ON LOSS OF PROFITS, DATA, FILES, USE, BUSINESS OPPORTUNITY OR CLAIMS OF THIRD PARTIES), AND WHETHER OR NOT THE PARTY HAS BEEN ADVISED OF THE POSSIBILITY OF SUCH DAMAGES. THIS LIMITATION APPLIES NOTWITHSTANDING ANY FAILURE OF ESSENTIAL PURPOSE OF ANY LIMITED REMEDY PROVIDED HEREIN.

10. Termination and Cancellation

10. 1. The License and all rights granted hereunder will continue until the end of the applicable period shown in Clause 5.1 above. Thereafter, this license will be terminated and all rights granted hereunder will cease.

10. 2. Licensor reserves the right to terminate the License in the event that payment is not received in full or if you breach the terms of this License.

11. General

11. 1. The License and the rights and obligations of the parties hereto shall be construed, interpreted and determined in accordance with the laws of the Federal Republic of Germany without reference to the stipulations of the CISG (United Nations Convention on Contracts for the International Sale of Goods) or to Germany´s choice-of-law principle.

11. 2. The parties acknowledge and agree that any controversies and disputes arising out of this License shall be decided exclusively by the courts of or having jurisdiction

for Heidelberg, Germany, as far as legally permissible.

11. 3. This License is solely for Licensor's and Licensee's benefit. It is not for the benefit of any other person or entity.

Questions? For questions on Copyright Clearance Center accounts or website issues please contact springernaturesupport@copyright.com or +1-855-239-3415 (toll free in the US) or +1-978-646-2777. For questions on Springer Nature licensing please visit https://www.springernature.com/gp/partners/rights-permissions-third-party-distribution

Other Conditions:

Version 1.4 - Dec 2022

Questions? customercare@copyright.com.
